# iIL13Pred: improved prediction of IL-13 inducing peptides using popular machine learning classifiers

**DOI:** 10.1186/s12859-023-05248-6

**Published:** 2023-04-11

**Authors:** Pooja Arora, Neha Periwal, Yash Goyal, Vikas Sood, Baljeet Kaur

**Affiliations:** 1 Department of Zoology, Hansraj College, University of Delhi, Delhi, India; 2 Department of Biochemistry, Jamia Hamdard, Delhi, India; 3 Department of Computer Science, Hansraj College, University of Delhi, Delhi, India

**Keywords:** Peptide prediction, Machine learning, IL-13, IL-13 peptides, mRMR, Feature selection

## Abstract

**Background:**

Inflammatory mediators play havoc in several diseases including the novel Coronavirus disease 2019 (COVID-19) and generally correlate with the severity of the disease. Interleukin-13 (IL-13), is a pleiotropic cytokine that is known to be associated with airway inflammation in asthma and reactive airway diseases, in neoplastic and autoimmune diseases. Interestingly, the recent association of IL-13 with COVID-19 severity has sparked interest in this cytokine. Therefore characterization of new molecules which can regulate IL-13 induction might lead to novel therapeutics.

**Results:**

Here, we present an improved prediction of IL-13-inducing peptides. The positive and negative datasets were obtained from a recent study (IL13Pred) and the Pfeature algorithm was used to compute features for the peptides. As compared to the state-of-the-art which used the regularization based feature selection technique (linear support vector classifier with the L1 penalty), we used a multivariate feature selection technique (minimum redundancy maximum relevance) to obtain non-redundant and highly relevant features. In the proposed study (improved IL-13 prediction (iIL13Pred)), the use of the mRMR feature selection method is instrumental in choosing the most discriminatory features of IL-13-inducing peptides with improved performance. We investigated seven common machine learning classifiers including Decision Tree, Gaussian Naïve Bayes, k-Nearest Neighbour, Logistic Regression, Support Vector Machine, Random Forest, and extreme gradient boosting to efficiently classify IL-13-inducing peptides. We report improved AUC, and MCC scores of 0.83 and 0.33 on validation data as compared to the current method.

**Conclusions:**

Extensive benchmarking experiments suggest that the proposed method (iIL13Pred) could provide improved performance metrics in terms of sensitivity, specificity, accuracy, the area under the curve - receiver operating characteristics (AUCROC) and Matthews correlation coefficient (MCC) than the existing state-of-the-art approach (IL13Pred) on the validation dataset and an external dataset comprising of experimentally validated IL-13-inducing peptides. Additionally, the experiments were performed with an increased number of experimentally validated training datasets to obtain a more robust model. A user-friendly web server (www.soodlab.com/iil13pred) is also designed to facilitate rapid screening of IL-13-inducing peptides.

**Supplementary Information:**

The online version contains supplementary material available at 10.1186/s12859-023-05248-6.

## Background

Cytokine storm, characterized by hyperproduction of pro-inflammatory cytokines such as IL-1, IL-2, IL-6, IFN-gamma, IL-13, IL-17, TNF-alpha etc. is considered one of the physio-pathological aspects correlated with the novel Coronavirus disease 2019 (COVID-19) disease severity [1–3]. Further in-vitro experimental studies validated by in-vivo data supported by insights obtained from single cell RNA-sequencing have demonstrated that inflammatory mediators in the serum of COVID-19 patients induced endothelial dysfunction and are highly correlated with COVID-19-associated endotheliopathy attesting to the pathological role of inflammatory cytokines in the disease [4, 5].

Interleukin (IL)-13 is one of the cytokines that has been recently associated as drivers of COVID-19 severity [6]. The role of IL13 in COVID-19 severity was also confirmed by other independent studies [7, 8]. IL-13 is a pleiotropic cytokine that is secreted by T-Helper 2 (Th-2) cells, basophils, mast cells, eosinophils, and natural killer cells [9]. Similar to IL-4, this cytokine plays role in Th-2-mediated immunity that includes responses to allergic reactions and parasitic infections. In fact, IL-13 causes class switching to IgG4 and IgE antibodies in naïve human B cells [10] and it is shown to play an indispensable role in the expulsion of gastrointestinal nematodes [11]. It is found to be an important mediator in airway inflammation seen in asthma and reactive airway diseases [12]. Independent and distinct from IL-4, IL-13 is also produced by Th1 and Th17 cells and is involved in adaptive immune responses including Th1 and Th17 inflammatory responses [13]. The fact that IL-13 is highly expressed in Hodgkin/Reed-Sternberg (H/RS) tumor cells [14] and blood cells of patients with autoimmune rheumatic diseases [15] indicates its role in the pathogenesis of neoplastic and autoimmune diseases. The role of IL-13 has been investigated in several bacterial and viral diseases. Elevated levels of IL-13 were observed in mice following *Chlamydia muridarum* infections [16]. It was further observed that IL-13 knock-out mice suffered from less disease severity, inflammation and bacterial load as compared to the wild-type mice infected with *Chlamydia muridarum*. Interestingly, elevated levels of IL-13 and IL-18 were also reported in patients with severe Dengue Hemorrhagic Fever suggesting that these cytokines play a critical role in the shift from Th1 to Th2 responses among them [17]. Another study reported that exogenous treatment of IL-13, IL-6, and IFN-g led to exacerbating pulmonary abnormality of enterovirus-infected mice [18].

Owing to the importance of IL-13 in COVID-19 severity and in regulating several vital biological processes, new molecules that can modulate the cytokine should be exploited. Recently, a tool (IL13Pred) was developed by Jain et al. [19] that aimed to classify IL-13-inducing peptides from the peptides that did not have the property to induce IL-13. The benchmark dataset included 343 experimentally validated IL-13-inducing peptides that were obtained from the immune epitope database [20]. However, further processing including the removal of duplicate peptides resulted in a list of 313 experimentally validated IL-13-inducing peptides. The negative datasets used by the authors included 2908 non-IL-13 inducing peptides and it was also obtained from the same database. Once the datasets were prepared, the authors then used the Pfeature algorithm to compute 9151 features of each peptide. The feature selection was then performed using the linear support vector classifier with the L1 penalty (SVC-L1) feature selection method which resulted in the identification of 95 relevant features. Once the features were identified, a decision tree-based algorithm was used to rank the features. The IL-13 prediction tool was then used to predict IL-13-inducing peptides.

**Fig. 1:**
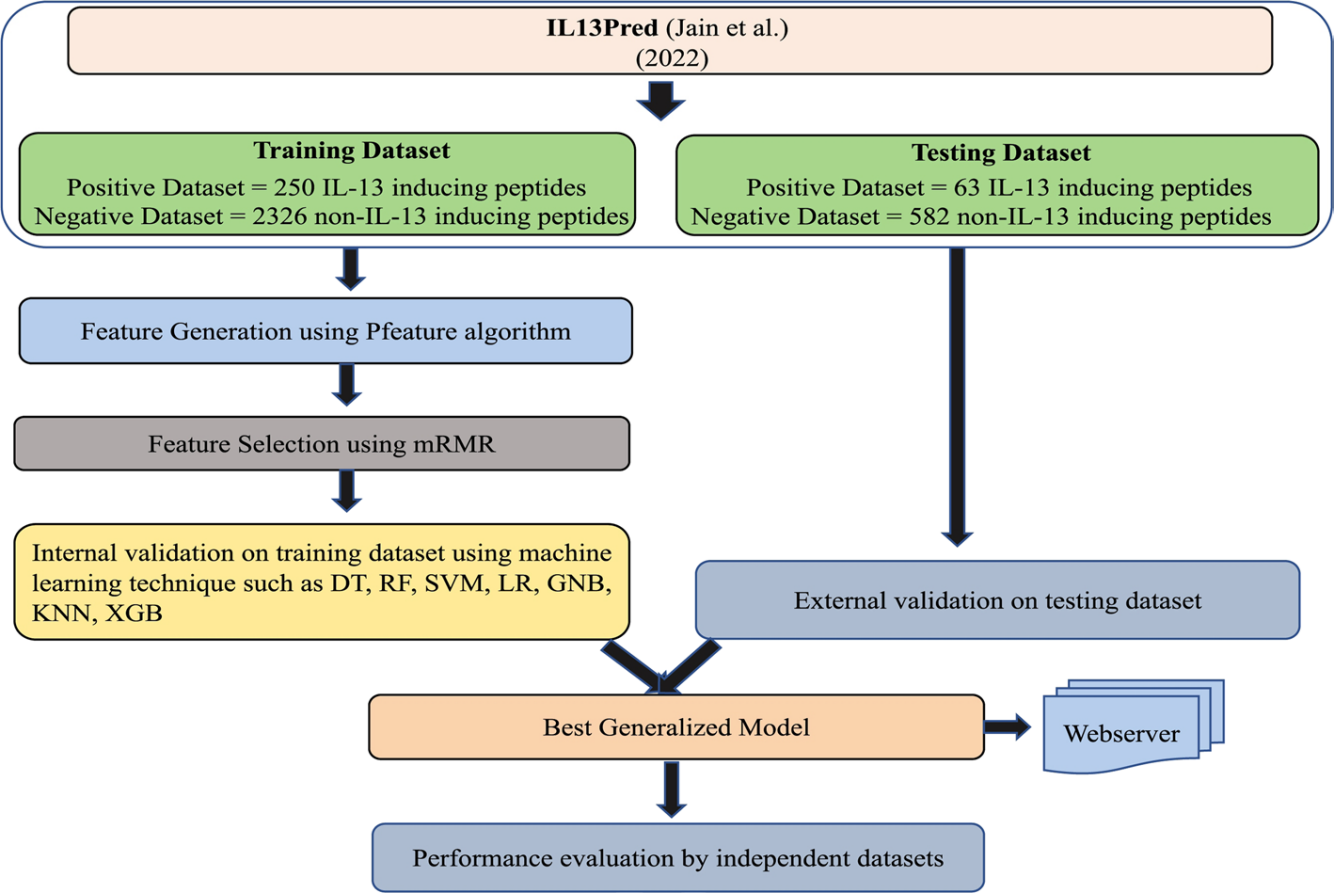
Overall architecture of the iIL13Pred design: The positive (IL-13 inducing peptides) and negative dataset (non-IL-13 inducing peptides) were obtained from IL13Pred (Jain et al. [19]).The positive and negative datasets were divided into 80:20 as training and testing data. The compositional features of Pfeature algorithm were used to compute features of IL-13 and non-IL-13 inducing peptides. Non-redundant and highly relevant feature selection tool mRMR was used to identify highly discriminatory and non-redundant features. Seven machine learning classifiers with five-fold internal cross validation was performed followed by an external validation on testing datasets. Best classifiers was then used to evaluate independent experimentally validated IL-13 inducing peptides. *Abbreviations:*IL-13, Interleukin-13; iIL13Pred, improved IL-13 prediction; mRMR, minimum redundancy maximum relevance; ML, Machine Learning; DT, Decision Tree; RF, Random Forest; SVM, Support Vector Machine; LR, Logistic Regression; GNB, Gaussian Naïve Bayes; KNN, *k*-Nearest Neighbour; XGB, eXtreme Gradient Boosting

In any machine learning (ML) system, the choice of the correct features is instrumental in building an effective and discriminative decision system. We wished to explore a more effective feature selection technique as compared to the SVC-L1 thereby leading to a further improvement of IL-13-inducing peptide prediction. Therefore, we propose an improved predictor of IL-13-inducing peptides which we named as iIL13Pred (improved IL-13 Prediction). The overall design of iIL13Pred is depicted in Fig. 1. All the positive and negative datasets were obtained from IL13Pred. Similar to the existing study, we also used the Pfeature algorithm to compute features of IL-13 and non-IL-13 inducing peptides. In our recent study (Periwal et al. manuscript communicated), we have observed the superiority of minimum redundancy maximum relevance (mRMR) feature selection over the SVC-L1 method. In comparison to SVC-L1, mRMR selects the non-redundant and highly relevant features that give a high performance when combined with diverse classification methods. Thus we implemented mRMR feature selection approach in this study.

Considering the importance of IL-13 in several biological processes, we aimed to build up an improved model of IL-13-inducing peptide prediction by relying on effective feature selection approach i.e. mRMR. In the proposed study, experimentally validated IL-13-inducing peptides (positive dataset) and non-IL-13-inducing peptides (negative dataset) were obtained from the IL-13 Pred tool [19]. A total of 9151 features for each peptide were generated from the compositional module of the Pfeature algorithm. Unlike IL-13 Pred, we used the mRMR feature selection method to identify highly discriminatory and non-redundant features. Ninety-five features were selected and were used to build machine learning classifiers as suggested in the baseline paper. Experiments with other feature set sizes were also performed. Similar to the IL13Pred, we also used seven machine learning classifiers including Decision Tree, Gaussian Naïve Bayes, k-Nearest Neighbour, Logistic Regression, Support Vector Machine, Random Forest, and eXtreme Gradient Boosting to efficiently classify IL-13-inducing peptides. We show that the improved IL-13 prediction (iIL13Pred) tool achieves better sensitivity and accuracy with nearly all the machine learning classifiers as compared to the existing method. Thus we propose that iIL13Pred can be used for efficient prediction of IL-13-inducing peptides.

## Material and Methods

### Benchmark datasets

Since IL13Pred is the most recent tool that aims to predict IL-13-inducing peptides, hence we used the same dataset in this study [19]. For the sake of comparison, all the datasets including the positive and negative datasets used in this study were obtained from the original study [19]. The positive dataset included 313 IL-13-inducing peptides whereas the negative dataset included 2908 non-IL-13-inducing peptides.

### Feature extraction

Accurate classification of peptide sequences relies on the generation of appropriate features. Similar to IL13Pred, we used the Pfeature algorithm to compute various features of both the IL-13 and non-IL-13 inducing peptides. The composition-based module of Pfeature algorithm was used to compute 9151 features for each peptide. Various descriptors of this module along with the number of features are described in Additional file 1: Table S1.

### Feature selection

For a given peptide sequence, the Pfeature algorithm generates 9151 features, most of which might be redundant in nature. Therefore, the selection of appropriate and highly relevant features is critical for the accurate performance of the machine learning classifier. Recently, we compared SVC-L1 and mRMR feature selection methods and observed the superiority of the mRMR feature selection method over the SVC-L1 method (Periwal et. al. manuscript communicated). Thus, in the current work, we used the mRMR feature selection method to extract the most relevant features.

#### mRMR feature selection

The presence of irrelevant and redundant features diminishes the generalization ability of a classification model. Hence, the identification of the features that are most relevant for classification is the major crucial step in building any machine learning model. The minimum redundancy maximum relevance (mRMR) feature selection method is one of the popular methods that are able to select the relevant features and remove the redundant features simultaneously.

The mRMR measure is denoted as

$$Max\left\{ {Relevance{-}Redundancy} \right\}$$

or

$$Max\left\{ {Relevance/Redundancy} \right\}$$

where

$$Relevance = \frac{1 }{{\left| {\text{S}} \right|}}\mathop \sum \limits_{i \in S} I\left( {i,c} \right)$$

$$Redundancy = \frac{1 }{{\left| {\text{S}} \right|^{2} }}\mathop \sum \limits_{i,j \in S} I\left( {i,j} \right)$$

*I*(*i, j*) denotes the mutual information among two features *i* and *j*. *I*(*i, c*) denotes the mutual information of the feature *i* with respect to class *c*. S is the set of features. The aim of mRMR is to choose the feature set where the mutual information amongst the features is minimized and the mutual information of the feature w.r.t the class is maximized. Given two features, *i* and *j*, with marginal probabilities, *p*(*i*) and *p*(*j*), and joint probability *p*(*i, j*), the mutual information *I*(*i, j*) is given by:

$$I\left( {i,j} \right) = \Sigma p\left( {i,j} \right)\log \frac{{p\left( {i, j} \right)}}{p\left( i \right)p\left( j \right)}$$

For the feature, *i* w.r.t. the class *c*, with marginal probabilities, *p*(*i*) and *p*(*c*), and joint probability *p*(*i, c*), the mutual information *I*(*i, c*) is given by:

$$I\left( {i,c} \right) = \Sigma p\left( {i,c} \right)\log \frac{{p\left( {i, c} \right)}}{p\left( i \right)p\left( c \right)}$$

### Classification models

Seven well-known classifiers are used in this study to build efficient decision models for the classification of IL-13 peptides: Decision Tree (DT), Gaussian Naïve Bayes (GNB), *k*-Nearest Neighbour (*K*NN), Logistic Regression (LR), Support Vector Machine (SVM), Random Forest (RF) and eXtreme Gradient Boosting (XGB). A decision tree-based classifier (DT) is a tree-based decision system where each branch represents the outcome of a test and the label at the leaf nodes identifies the class that is decided upon by the classifier. The Gaussian Naïve Bayes classifier (GNB) is based on the Bayes theorem and follows Gaussian distribution while supporting continuous data. The *K* nearest neighbour classifier (*K*NN) takes into consideration *K* data instances that are closest to the test sample and attributes the majority class to the test sample. Logistic Regression (LR) predicts the probability of the target variable using the S-shaped logistic function where the coefficients of the logistic regression algorithm are estimated using the maximum-likelihood estimation. The support vector machine classifier (SVM) determines an optimum decision boundary that maximizes the margin between the hyperplanes passing through the support vectors of the two classes. Various kernel functions facilitate the realization of non-linear decision boundaries in an SVM classifier. A random forest algorithm (RF) is a collection of decision trees. Each participating tree is formed from a different training set and hence each has a unique performance. Based on the collective decision of the participating decision trees, the final decision of the random forest is reported. RF exhibits improved performance as compared to when only a single decision tree is modelled. The eXtreme Gradient Boosting (XGB) classifier is an ensemble approach based on the gradient boosting decision tree technique where the errors of the existing models are improved by newer models. It is a highly efficient and scalable method that avoids overfitting and offers high performance of unseen and novel data.

### Internal cross-validation and external validation

To train, test, and evaluate our prediction models, we used a similar approach as used by Jain et al. [19]. The dataset was split into a ratio of 80:20 to obtain training and validation datasets. After the data was split, our training dataset comprised 250 positive and 2326 negative peptides whereas the validation dataset comprised 63 positive and 582 negative peptides. We used the 5-fold cross-validation and external validation technique. The parameter range for all the classifiers used for internal validation has been provided in Additional file 2: Table S2. The best parameters that were obtained during the 5-fold cross-validation were then used to test the external validation dataset. Various common performance metrics used for the evaluation of the classifiers included sensitivity, specificity, accuracy, area under the curve - receiver operating characteristics (AUCROC) and Mathews correlation coefficient (MCC).

### Evaluation parameters

The most commonly used threshold-dependent and independent parameters including sensitivity, specificity accuracy, and AUCROC were used in this study. These parameters can be defined as follows:

$${\text{Sensitivity}} = {\text{TP}}/\left( {{\text{TP}} + {\text{FN}}} \right)$$

$${\text{Specificity}} = {\text{TN}}/\left( {{\text{TN}} + {\text{FP}}} \right)$$

$${\text{Accuracy}} = \left( {{\text{TP}} + {\text{TN}}} \right)/\left( {{\text{TP}} + {\text{FP}} + {\text{TN}} + {\text{FN}}} \right)$$

TP = True Positive, FP = False Positive.

TN = True Negative, FN = False Negative.

Area under the curve - Receiver Operating Characteristics (AUCROC) explains the efficiency of a classification model. It is calculated in threshold independent manner. Higher the AUC, the better the model in differentiating the positive and negative class. The MCC is the preferred performance metric in case of unbalanced data and gives a high score if most of the positive and negative predictions are correct [21].

$${\text{MCC}} = \left( {\left( {{\text{TP}}*{\text{TN}}} \right) {-} \left( {{\text{FP}}*{\text{FN}}} \right)} \right)/\sqrt {\left( {{\text{TP}} + {\text{FP}}} \right) \left( {{\text{TP}} + {\text{FN}}} \right)\left( {{\text{TN}} + {\text{FP}}} \right)\left( {{\text{TN}} + {\text{FN}}} \right)}$$

### Design of web-based prediction tool

An intuitive web application was designed and developed to facilitate the prediction of IL-13 peptides. The application allows users to input a peptide sequence, which is then processed by a machine-learning algorithm. This tool predicts the IL-13 induction potential of the given peptide. The development of the application involved the use of multiple web technologies coupled with a proficient machine-learning algorithm. Flask, which is a leading Python web framework, was used to host this web application on an Amazon Web Services (AWS) instance.

To create an interactive front end for the application, we used HTML, CSS, and JavaScript. HTML, which is also known as the building block of the web, was used to structure web pages. CSS, a design framework used for adding design to the web pages, and finally JavaScript, the scripting language of the web, was used to add interactivity to the web pages like form validations, drop-down menus, and other interactive elements that allowed users to interact with the machine learning algorithm. The next step involved integrating the machine learning model at the backend with the front end of the web application. This model was trained on a large dataset, and the results were saved in a file on the server to facilitate further predictions. A python program integrated with our Flask application could interact with the machine learning model and pass the generated predictions to the front end of the web application.

Apart from predicting the IL13-inducing potential of a given peptide, additional functionalities of the webserver included *design* and *peptide scan*. The design module in iIL13Pred mutates a single amino acid of the peptide sequence at a time and then predicts the IL-13 induction potential of the resulting mutants. The protein scan module of iIL13Pred generates all the possible overlapping peptides and predicts the IL-13 induction potential of all the resulting peptides.

## Results

### Feature extraction and selection

**Fig. 2:**
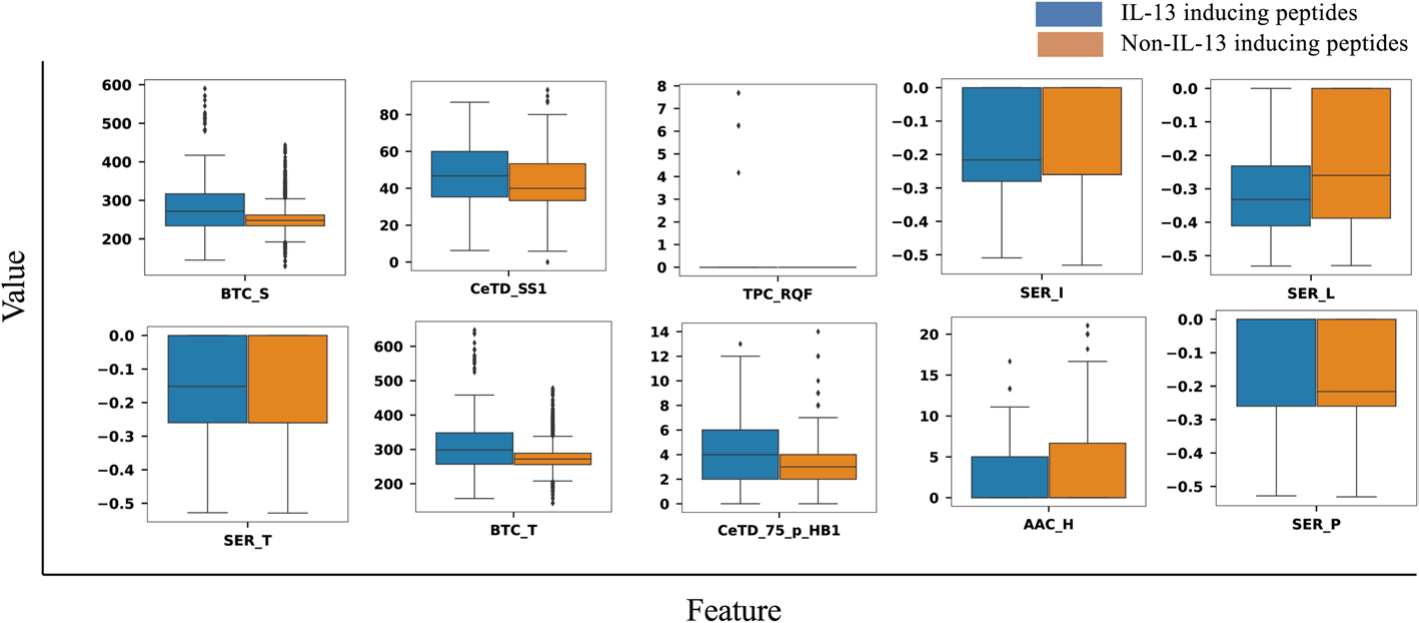
The boxplot representation of the top 10 features selected by the mRMR feature selection method indicates their discriminatory nature: The top 10 features selected by the mRMR feature selection method were plotted for IL-13 inducing and non-IL-13 inducing peptides. The features were found to be highly discriminatory as seen in the box plot of the top 10 features. *Abbreviations:* mRMR, minimum redundancy maximum relevance; BTC_S, Composition of Single bonds; CeTD_SS1, Composition of group 1 residues for secondary structure attribute; TPC_RQF, Composition of Arginine–Glutamine–Phenylalanine tripeptide; SER_I, Shannon entropy for residue Isoleucine; SER_L, Shannon entropy for residue Leucine; SER_T, Shannon entropy for residue Threonine; BTC_T, Composition of total bonds; CeTD_75_p_HB1, Number of group 1 residues for hydrophobicity present in 75% quartile; AAC_H, Amino acid composition of Histidine; SER_P, Shannon entropy for residue Proline

Once the positive and negative datasets were curated, the Pfeature algorithm was used to compute 9151 features of each peptide sequence. We then used the mRMR feature selection method to identify the most relevant features. We performed experiments with the top 10, 20, 30,…95 features to build the machine learning models similar to the methodology used by Jain et al. [19]. For the sake of fair comparison with the state of the art, the results with 10, 20, 30,…95 features are presented in the Additional file 3: Table S3. To visually assert the discriminatory nature of the top ten features, we have plotted the box plots for each in Fig. 2. It can be observed that the features selected by mRMR tend to be highly discriminatory in nature. This strengthens the role of the features chosen by the mRMR method and indicates that these are indeed effective in the improved classification of IL-13-inducing peptides.

### Machine-learning based prediction models

**Fig. 3:**
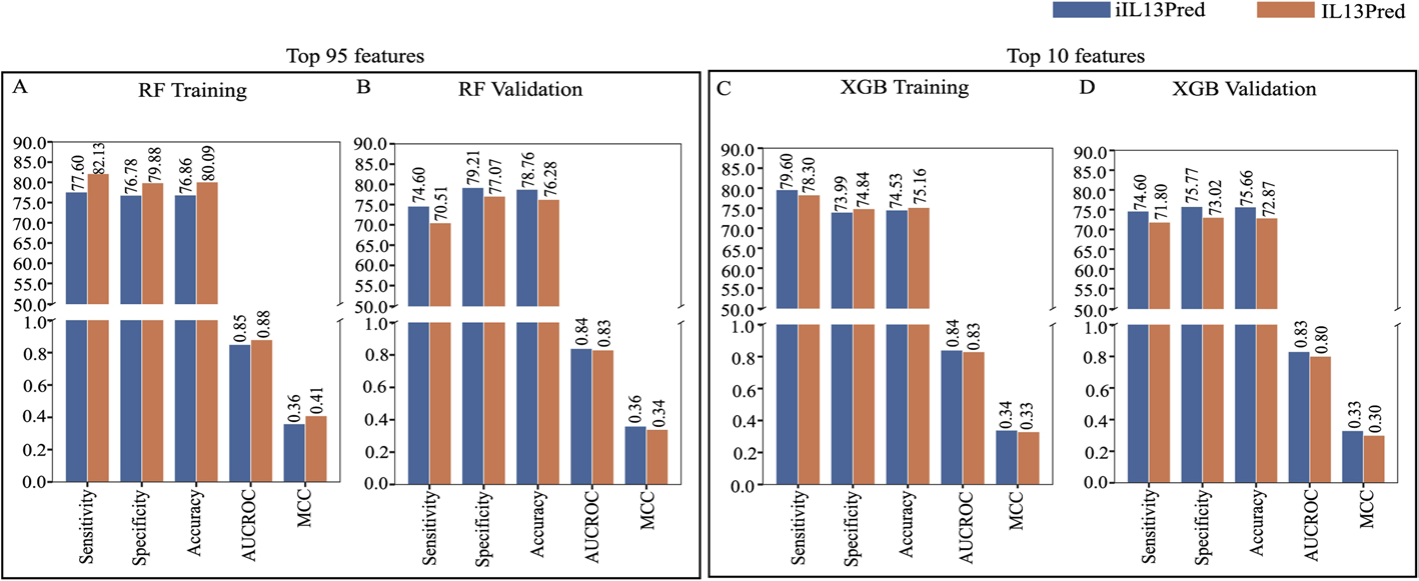
Comparison of performance metrics of iIL13Pred and IL13Pred: **A**, **B** Comparison of performance metrics of RF classifier on top 95 features in training and validation datasets respectively in iIL13Pred and IL13Pred and **C**, **D** Comparison of performance metrics of XGB classifier on top 10 features in training and validation data respectively in iIL13Pred and IL13Pred. *Abbreviations:* iIL13Pred, improved IL-13 prediction; IL13Pred, IL-13 prediction; RF, Random Forest; XGB, eXtreme Gradient Boosting; AUCROC, Area under the curve - Receiver Operating Characteristics; MCC, Matthews correlation coefficient

**Fig. 4:**
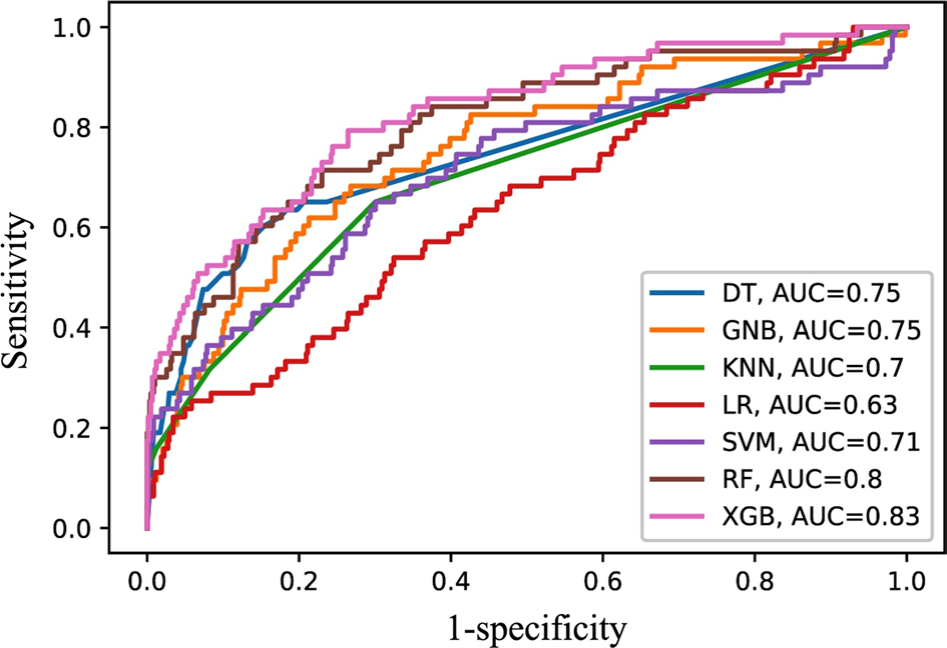
ROC curve of seven machine learning models using top 10 features on validation data: The model built using XGB classifier (represented by pink solid line) shows the best AUC followed by RF classifier. The X-axis represents the false positive rate i.e. 1-Specificity while Y-axis represents the true positive rate i.e. Sensitivity. *Abbreviations*: ROC, Receiver Operating Characteristics; DT, Decision Tree; RF, Random Forest; SVM, Support Vector Machine; LR, Logistic Regression; GNB, Gaussian Naïve Bayes; KNN, *k*-Nearest Neighbour; XGB, eXtreme Gradient Boosting

**Fig. 5:**
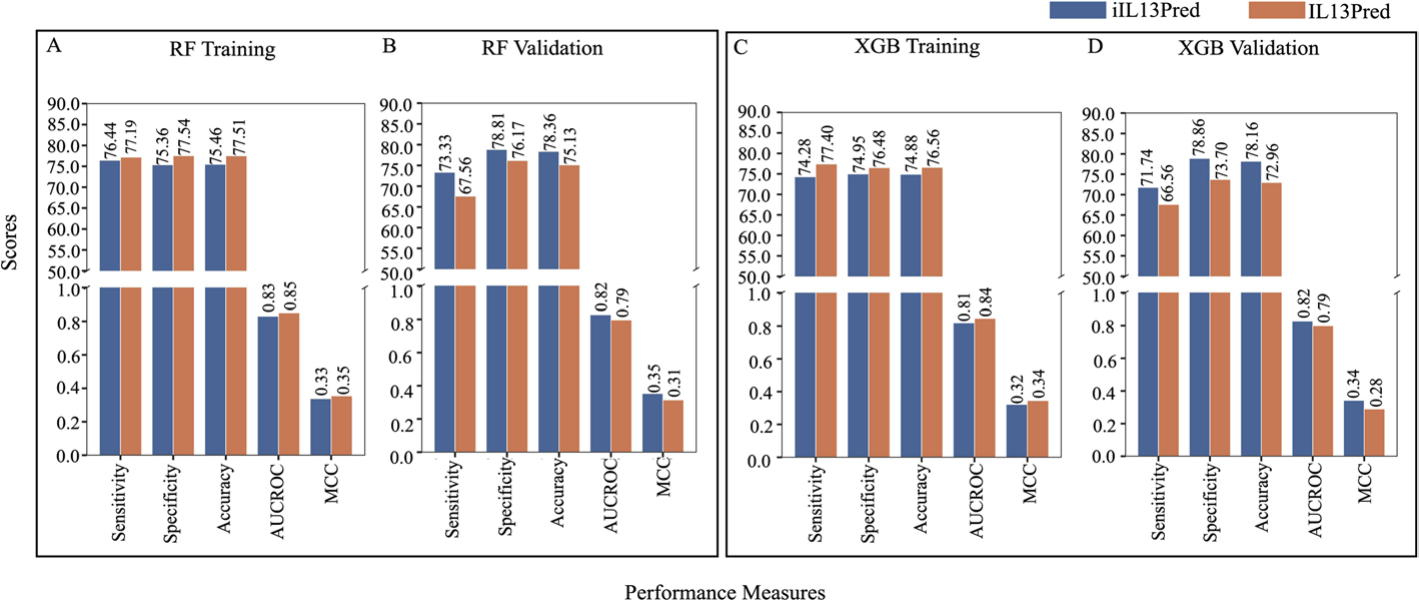
Comparison of the averages of all the performance measures of the features (10–95) for various ML parameters on training and validation datasets for **A** Random Forest training **B** Random Forest validation, **C** XGB Training and **D** XGB validation classifiers

We used seven different machine learning classifiers i.e. Decision Tree, Gaussian Naïve Bayes, *k*-Nearest Neighbour, Logistic Regression, Support Vector Machine, Random Forest and eXtreme Gradient Boosting. The best parameters obtained from the 5-fold internal cross-validation were then used to calculate the average sensitivity, specificity, accuracy, AUCROC, and MCC for the validation data. Similar to the methodology that was used in the existing method (IL13Pred), we evaluated several machine learning classifiers on the top 10, 20, 30,…95 features obtained using the mRMR feature selection method. A comparison of both the tools (proposed and the state of the art) revealed the following:

i. The current method (IL13Pred) reported that among the seven ML classifiers, the RF classifier performed the best on 95 features with an AUCROC of 0.88 on training and 0.83 on validation data. We used the mRMR feature selection method and report an AUCROC score of 0.85 on training and 0.84 in the case of validation dataset with RF classifier. MCC is known to be the preferred metric in case of an unbalanced data [21]. We further report an improved MCC of 0.36 as compared to 0.34 for the validation data obtained using the RF classifier (Fig. 3A–B and Table 1).
ii. In addition to reporting the performance on the top 95 features, the current study [19] further shows that the XGB classifier outperformed all other classifiers with AUCROC of 0.83 and 0.80 in training and validation datasets respectively using the top 10 features (Table 2). Using similar approaches, we also performed the experiments with our top 10 features and show an improvement in the results. We report an improved AUCROC of 0.84 and 0.83 in training and validation datasets respectively with the XGB classifier. Additionally, we report improved MCC scores of 0.34 and 0.33 on training and validation datasets as compared to the IL13Pred which reported MCC scores of 0.33 and 0.30 for testing and validation data respectively (Fig. 3C–D and Table 2). The data suggest that our models are more efficient in classifying IL-13 inducing peptides as compared to the existing method. The selection of the most discriminatory features using the mRMR method is instrumental in the improved performance of all feature sets and all classifiers.
iii. AUCROC indirectly assesses the performance of the classifier. A model with a larger value of AUC is usually considered as a better classifier in comparison to the one with a smaller value. The AUC score of seven machine learning models on the top 10 features was plotted in validation data. It was observed that XGB was found to be a better classifier followed by RF classifier. The supremacy of the XGB classifier over the other classifiers is shown in the AUCROC plot (Fig. 4).
iv. In order to further compare our models with IL13Pred, we performed all the experiments on 10, 20, 30, 40,…95 features. We show that our classifiers were superior in most of the cases as compared to the existing method (Additional file 3: Table S3) pointing toward the effectiveness of the features selected using mRMR feature selection technique. An improved average sensitivity, specificity, accuracy, AUCROC, and MCC was reported for 10, 20, 30,40,…95 features using DT, GNB, KNN, and SVC classifiers (Additional file 4: Fig. S1). In the case of LR, RF and XGB classifiers, an improved average sensitivity, specificity, accuracy, AUCROC and MCC was reported for 10,20,30,40, …95 features for the validation data (Fig. 5 and Additional file 4: Fig. S1). We observe a marginal dip in the training performance. This is attributed to the generalized performance of the proposed decision model. In ML, those models are preferred which although may have less training performance as they are better generalized and give improved validation results. This is affirmed by the higher performance of the decision models on the validation dataset.

**Table 1:** Performance metrics of seven machine learning models for prediction of IL-13 inducing peptides on the top 95 features via mRMR (iIL13Pred) and its comparison with the state of the art (IL13Pred)

Classifier	Dataset	Sensitivity		Specificity		Accuracy		AUCROC		MCC	
		Proposed study	Jain et al. [19]	Proposed study	Jain et al. [19]	Proposed study	Jain et al. [19]	Proposed study	Jain et al. [19]	Proposed study	Jain et al. [19]
DT	Training	**70.80**	65.96	**76.09**	66.38	**75.57**	66.34	**0.76**	0.70	**0.31**	0.19
	Validation	**63.49**	51.28	**74.40**	66.31	**73.33**	64.50	**0.71**	0.60	**0.25**	0.12
GNB	Training	**67.12**	63.83	**80.13**	79.58	**78.88**	78.14	**0.81**	0.78	**0.33**	0.29
	Validation	**50.79**	38.46	**78.69**	77.43	**75.97**	72.71	**0.71**	0.61	**0.20**	0.12
KNN	Training	**66.00**	57.02	**69.91**	65.61	**69.53**	64.83	**0.73**	0.64	**0.23**	0.14
	Validation	**61.90**	50.00	**81.79**	69.31	**79.84**	66.98	**0.74**	0.62	**0.31**	0.13
LR	Training	70.00	**73.62**	70.00	**73.99**	70.00	**73.95**	0.77	**0.83**	0.25	0.30
	Validation	**61.90**	58.97	**73.37**	68.25	**72.25**	67.13	**0.71**	0.68	**0.23**	0.19
SVC	Training	**74.00**	72.34	**72.14**	71.25	**72.32**	71.35	**0.82**	0.79	**0.29**	0.27
	Validation	**68.25**	51.28	**71.31**	68.08	**71.00**	66.05	**0.80**	0.62	**0.25**	0.13
RF	Training	77.60	**82.13**	76.78	**79.88**	76.86	**80.09**	0.85	**0.88**	0.36	**0.41**
	Validation	**74.60**	70.51	**79.21**	77.07	**78.76**	76.28	**0.84**	0.83	**0.36**	0.34
XGB	Training	**77.27**	73.62	72.27	**76.59**	72.76	**76.32**	0.83	**0.84**	0.31	**0.32**
	Validation	**73.02**	69.23	**79.73**	73.19	**79.07**	72.71	**0.81**	0.80	**0.36**	0.30

The higher values are highlighted in bold

**Table 2:** Performance metrics of seven machine learning models for prediction of IL-13 inducing peptides on the top 10 features via mRMR (iIL13Pred) and its comparison with the state of the art (IL13Pred)

Classifier	Dataset	Sensitivity		Specificity		Accuracy		AUCROC		MCC	
		Proposed study	Jain et al. [19]	Proposed study	Jain et al. [19]	Proposed study	Jain et al. [19]	Proposed study	Jain et al. [19]	Proposed study	Jain et al. [19]
DT	Training	64.80	**69.36**	**72.18**	69.46	**71.47**	69.45	0.72	**0.74**	0.24	0.24
	Validation	**65.08**	60.26	**76.29**	71.43	**75.19**	70.08	**0.75**	0.72	**0.27**	0.22
GNB	Training	**72.00**	71.06	**70.98**	68.18	**71.08**	68.44	**0.79**	0.74	**0.29**	0.24
	Validation	**68.25**	64.10	**72.34**	66.31	**71.94**	66.05	**0.75**	0.73	**0.26**	0.21
KNN	Training	**68.40**	65.11	**66.17**	56.47	**66.38**	57.26	**0.72**	0.64	**0.21**	0.13
	Validation	**65.08**	60.26	**69.93**	55.73	**69.46**	56.28	**0.70**	0.61	**0.22**	0.11
LR	Training	**66.00**	64.26	**62.59**	61.85	**62.92**	62.07	**0.67**	0.67	**0.17**	0.15
	Validation	**60.32**	56.41	**62.03**	59.61	**61.86**	59.23	**0.63**	0.63	**0.14**	0.11
SVC	Training	**60.40**	54.47	**74.46**	70.70	**73.10**	69.22	**0.74**	0.67	**0.23**	0.16
	Validation	**49.21**	46.15	**79.04**	74.25	**76.12**	70.85	**0.71**	0.64	**0.20**	0.15
RF	Training	**76.40**	74.47	75.02	**76.34**	75.16	**76.17**	0.84	0.83	**0.34**	0.33
	Validation	**68.25**	64.10	**78.18**	75.13	**77.21**	73.80	**0.80**	0.77	**0.31**	0.28
XGB	Training	**79.60**	78.30	73.99	**74.84**	74.53	**75.16**	**0.84**	0.83	**0.34**	0.33
	Validation	**74.60**	71.80	**75.77**	73.02	**75.66**	72.87	**0.83**	0.80	**0.33**	0.30

The higher values are highlighted in bold

### Case study 1: prediction of IL-13 inducing peptides from SARS-CoV-2 proteins

Recently, IL-13 was reported to be associated with the severity of COVID-19. Therefore Jain et al. [19] obtained multiple sequences of SARS-CoV-2 and used the protein scan module of their web server to identify IL-13-inducing peptides encoded by SARS-CoV-2 spike protein. The authors retrieved several SARS-CoV-2 sequences from five different countries and predicted 213 IL-13-inducing peptides. The authors further identified ten potential IL-13-inducing peptides in the SARS-CoV-2 spike protein. In order to compare the performance of our model with IL13Pred, we tested the same ten major IL-13-inducing peptides from the spike protein of SARS-CoV-2 and found that our model predicted a reduced probability of these peptides to induce IL-13 as compared to the current method (Table 3).

**Table 3:** Case study 1: potential IL-13 inducing peptides from SARS-CoV-2 spike protein

S.No	Peptide sequence	Proposed study (probability)	Jain et al. [19] (probability)
1	ELDSFKEELDKYFKN	0.06	**0.39**
2	LLTDEMIAQYTSALL	0.03	**0.32**
3	KQGNFKNLREFVFKN	0.05	**0.30**
4	EIDRLNEVAKNLNES	0.04	**0.30**
5	VNIQKEIDRLNEVAK	0.06	**0.27**
6	LYRLFRKSNLKPFER	0.22	**0.25**
7	NIQKEIDRLNEVAKN	0.11	**0.23**
8	KSTNLVKNKCVNFNF	0.12	**0.22**
9	VLTESNKKFLPFQQF	0.02	**0.22**
10	IQKEIDRLNEVAKNL	0.14	**0.22**

The higher values are highlighted in bold

### Case study 2: prediction of IL-13 inducing peptides from spike protein of SARS-CoV-2 and its variants

Since the start of the SARS-CoV-2 pandemic, the virus has been continuously mutating. The chance mutation among the viral proteins might lead to the possibility of gain or loss of functions. Therefore, Jain et al. [19] attempted to study the role of mutations in the IL-13 induction. The authors obtained the reference sequence of spike protein and then engineered highly prevalent mutations from SARS-CoV-2 variants including Alpha (B.1.1.7), Beta (B.1.351), and Delta (B.1.617.2) in the reference sequence. The authors then predicted the IL-13-inducing ability in these peptides. We performed similar experiments with our tool and observed that results obtained from IL13Pred were superior as compared to our results suggesting that our tool lagged behind in predicting IL-13-inducing peptides from variants of the spike protein of SARS-CoV-2 (Table 4). It was observed that though our tool resulted in better results on the validation dataset, it did not perform well on the SARS-CoV-2 peptide data. We concluded that since none of the above-mentioned IL-13-inducing SARS-CoV-2 peptides were experimentally validated, so we sought to investigate our tool on an unseen external set comprising of experimentally validated dataset.

**Table 4:** Case study 2: potential IL-13 inducing peptides from SARS-CoV-2 wild-type and mutated spike protein

SARS-CoV-2 variants	Mutation	Reference peptide	Mutated peptide	Score_R (probability)		Score_M (probability)	
				Proposed study	Jain et al. [19]	Proposed study	Jain et al. [19]
Alpha (B.1.1.7)	A570D	NKKFLPFQQFGRDIA	NKKFLPFQQFGRDID	0.03	**0.05**	0.04	**0.11**
		KFLPFQQFGRDIADT	KFLPFQQFGRDIDDT	**0.04**	0.03	0.03	**0.06**
	T716I	SNNSIAIPTNFTISV	SNNSIAIPINFTISV	0.01	**0.04**	**0.07**	0.06
	S980A	NFGAISSVLNDILSR	NFGAISSVLNDILAR	0.02	**0.04**	0.02	**0.06**
		GAISSVLNDILSRLD	GAISSVLNDILARLD	**0.04**	0.03	0.06	**0.08**
		VLNDILSRLDKVEAE	VLNDILARLDKVEAE	**0.07**	0.03	0.04	**0.07**
		LNDILSRLDKVEAEV	LNDILARLDKVEAEV	**0.07**	0.05	0.04	**0.08**
		NDILSRLDKVEAEVQ	NDILARLDKVEAEVQ	**0.05**	0.04	0.05	**0.09**
	D1118H	TQRNFYEPQIITTDN	TQRNFYEPQIITTHN	0.01	**0.06**	0.01	**0.04**
		QRNFYEPQIITTDNT	QRNFYEPQIITTHNT	0.01	**0.07**	0.01	**0.03**
		YEPQIITTDNTFVSG	YEPQIITTHNTFVSG	0.01	**0.04**	0.01	**0.06**
Beta (B.1.351)	L18F	LVLLPLVSSQCVNLT	LVLLPLVSSQCVNFT	0.05	**0.06**	**0.05**	0.04
		LLPLVSSQCVNLTTR	LLPLVSSQCVNFTTR	0.01	**0.07**	0.01	**0.02**
	D80A	VSGTNGTKRFDNPVL	VSGTNGTKRFANPVL	0.01	**0.05**	0.01	**0.12**
		GTNGTKRFDNPVLPF	GTNGTKRFANPVLPF	0.01	**0.02**	0.01	**0.07**
		SGTNGTKRFDNPVLP	SGTNGTKRFANPVLP	0.01	**0.09**	0.01	**0.02**
Delta (B.1.617.2	T19R	LVLLPLVSSQCVNLT	LVLLPLVSSQCVNLR	0.05	**0.06**	0.04	0.04
	R158G	FRVYSSANNCTFEYV	FGVYSSANNCTFEYV	0.02	**0.03**	0.02	**0.06**
	P618R	TQTNSPRRARSVASQ	TQTNSRRRARSVASQ	0.01	**0.02**	0.02	**0.08**
		QTNSPRRARSVASQS	QTNSRRRARSVASQS	0.02	0.02	0.02	**0.08**
	D950N	DSLSSTASALGKLQD	DSLSSTASALGKLQN	0.05	0.05	0.04	**0.06**

The higher values are highlighted in bold

### Case study 3: prediction of experimentally validated IL-13 inducing peptides

Since in both the above-mentioned case studies we were testing our tool on SARS-CoV-2 data which was not experimentally validated at all, we could not rely on the prediction of our models. To validate the prediction efficiency of our tool, we sought to test our model on the experimentally validated IL-13-inducing peptides. In the current study, all the experimentally validated IL-13-inducing peptides were obtained from the immune epitope database that probably may have been accessed in August–September 2021 [19]. Since the database is regularly updated, there might have been an addition of some more experimentally validated IL-13-inducing peptides. We accessed the immune database in May 2022 and obtained some additional experimentally validated IL-13-inducing peptides (Table 5) that were not included by Jain et al. [19]. Since these peptides were characterized experimentally, hence we sought to benchmark both the tools (IL13Pred and iIL13Pred) on this dataset. The results thus obtained revealed that out of a total of 68 experimentally validated IL-13-inducing peptides, our tool predicted 37 peptides to be IL-13 inducers with greater probability as compared to the IL13Pred which predicted 19 peptides to be IL-13 inducers with greater probability. It was also observed that three peptides were predicted to be IL-13 inducers with the same probability using both tools. Out of the 68 experimentally validated IL-13-inducing peptides, our tool misclassified only 13% of the peptides (n = 9) as non-IL-13 inducers whereas the current method (IL13Pred) misclassified 16% of the peptides (n = 11) as non-IL-13 inducers. Additionally, the average prediction probability of our tool was (0.424) is greater as compared to the state-of-the-art (0.389) pointing towards the supremacy of our tool.

**Table 5:** Case study 3: prediction of experimentally validated IL-13 inducing peptides

S.No	Peptide sequence	Proposed study	Proposed study	Jain et al. [19]	Jain et al. [19]
		Score	Prediction	Score	Prediction
1	KKGELALFYLQEQINHFEEKPTKEMKDKIVAEMDTI	0.95	IL-13	**0.98**	IL-13
2	GYFADPKDPHKFYICSNWEAVHKDCPGNTRWNEDEETCT	**0.94**	IL-13	0.92	IL-13
3	PDEVRRMMAEIDTDGDGFISFDEFTDFARANRGLVKDVSKIF	**0.94**	IL-13	0.92	IL-13
4	TNACSINGNAPAEIDLRQMRTVTPIRMQGGCGSCWAFSGVA	**0.94**	IL-13	0.92	IL-13
5	AAEDTPQDIADRERIFKRFDTNGDGKISSSELGDALKTLGSVTP	0.94	IL-13	**0.96**	IL-13
6	PEGFPFKYVKDRVDEVDHTNFKYNYSVIEGGPIGDTLEKISNEIK	**0.94**	IL-13	0.86	IL-13
7	FGISNYCQIYPPNANKIREALAQPQRYCR	0.94*	IL-13	0.94*	IL-13
8	ATESAYLAYRNQSLDLAEQELVDCASQHGCHGDTIPRGIEYIQ	**0.93**	IL-13	0.90	IL-13
9	DTPQDIADRERGGSFDTNGDGKISSGGSTDGDGFISFDEFTDFARANRGLVKDV	0.93	IL-13	**0.98**	IL-13
10	LHLSEQYKELEKTKSKELKEQILRELTIGENFMKGAL	0.93	IL-13	**0.95**	IL-13
11	EVDVPGIDPNACHYMKCPLVKGQQYDIKYTWIVPKIAPKSEN	0.93*	IL-13	0.93*	IL-13
12	REQSCRRPNAQRFGISNYCQIYPPNVNKIREALAQTH	**0.93**	IL-13	0.90	IL-13
13	WMHHNMDLI	**0.93**	IL-13	0.53	IL-13
14	KLQCVDLHV	0.92	IL-13	**0.96**	IL-13
15	LFPKVAPQAISSVENIEGNGGPGTIKKISFPEGFPFKYVKDRVDE	**0.92**	IL-13	0.90	IL-13
16	VHDDVVSMEYDLAYKLGDLHPNTHVISDIQDFVVEL	0.92	IL-13	**0.96**	IL-13
17	LSVGWISGQY	**0.92**	IL-13	0.31	IL-13
18	DAEFRHDSGYEVHHQKLVFFAEDVGSNKGAIIGLMVGGVVIA	**0.91**	IL-13	0.82	IL-13
19	IYSTVASSL	0.90	IL-13	**0.98**	IL-13
20	NYEEAQTLSK	**0.85**	IL-13	0.82	IL-13
21	AKFVAAWTLKAAA	**0.78**	IL-13	0.72	IL-13
22	PITAKAIAASVG	**0.55**	IL-13	0.36	IL-13
23	EAALAAFAKIAE	0.54	IL-13	**0.64**	IL-13
24	EAALAKFAAIAE	0.54	IL-13	**0.66**	IL-13
25	EAALKAFAAIAE	0.54	IL-13	**0.59**	IL-13
26	LDVVCAMIEGAQG	**0.53**	IL-13	0.49	IL-13
27	SLGWATLVGEITAGNLLHTR	**0.53**	IL-13	0.27	IL-13
28	PRFIAVGYVDDTE	**0.51**	IL-13	0.11	IL-13
29	YDGSVVAINP	**0.46**	IL-13	0.24	IL-13
30	GTCLESLRRYLELGKERL	**0.40**	IL-13	0.38	IL-13
31	LVRYWISAFP	0.34	IL-13	**0.58**	IL-13
32	GPTHLFQPSLVLDMAKVLLD	0.30	IL-13	**0.35**	IL-13
33	IVDTISDFRAAIANYHYDAD	**0.29**	IL-13	0.26	IL-13
34	QNGRWISRDP	**0.27**	IL-13	0.21	IL-13
35	NNSYECDIPIGAGICASYQ	**0.24**	IL-13	0.17	IL-13
36	FARQAVWLRE	0.24	IL-13	**0.33**	IL-13
37	YTTGAVRQIFGDYKTTICGK	**0.23**	IL-13	0.08	IL-13
38	AENPRMEPRARWMEREGPEYW	**0.22**	IL-13	0.07	IL-13
39	IYNRNIVNRL	**0.20**	IL-13	0.19	IL-13
40	WNRKRISNCVADYSVLYNS	**0.20**	IL-13	0.13	IL-13
41	MEVGWYRSSFSRVVHLYRNGK	**0.19**	IL-13	0.10	IL-13
42	QAPEYRGRTELLKDAIGEGKVTLRI	**0.18**	IL-13	0.09	IL-13
43	GYKDGNEYI	0.18	IL-13	**0.23**	IL-13
44	NKIQDKVTIDGY	**0.18**	IL-13	0.06	IL-13
45	AALALLLLDRLNQLE	**0.16**	IL-13	0.07	IL-13
46	FEELIKFSFHTNVLEDNIGY	0.15	IL-13	**0.20**	IL-13
47	LRHNPGGPSSAVPLLLSYFQ	**0.14**	IL-13	0.07	IL-13
48	MESGEWVIKE	**0.12**	IL-13	0.10	IL-13
49	SGIPYIISYLHPGNTILHVD	0.10	IL-13	**0.11**	IL-13
50	SGIPYVISYLHPGNTVMHVD	**0.10**	IL-13	0.05	Non-IL-13
51	HWFVTQRNFYEPQII	0.09	IL-13	**0.11**	IL-13
52	HPGNTILHVDTIYNRPSNTT	**0.09**	IL-13	0.07	IL-13
53	VGGNYNYLYRLFRKSNLKP	0.09	IL-13	**0.21**	IL-13
54	FNNFTVSFWLRVPKVSASHLE	**0.09**	IL-13	0.08	IL-13
55	MEVGWYRSPFSRVVHLYRNGK	**0.08**	IL-13	0.07	IL-13
56	DESTESETEQAF	**0.07**	IL-13	0.04	Non-IL-13
57	MEVGWYRPPFSRVVHLYRNGK	0.07*	IL-13	0.07*	IL-13
58	HSLGKLLGRPDKF	0.07	IL-13	**0.12**	IL-13
59	AGFKGEQGPKGEP	**0.06**	IL-13	0.03	Non-IL-13
60	HSLGKWLGHPDKF	0.06	Non-IL-13	0.04	Non-IL-13
61	ISQAVHAAHAEINEAGR	0.05	Non-IL-13	0.03	Non-IL-13
62	NCTFEYVSQPFLMDL	0.04	Non-IL-13	0.05	Non-IL-13
63	NAGFNSNRANSSRSS	0.03	Non-IL-13	0.02	Non-IL-13
64	QYIKANSKFIGITEL	0.03	Non-IL-13	0.02	Non-IL-13
65	VHFFKNIVTPRTPPPSQGKGR	0.03	Non-IL-13	0.08	IL-13
66	KIYNRNIVNRLLGD	0.02	Non-IL-13	0.05	Non-IL-13
67	NTWTTCQSIAFPSK	0.01	Non-IL-13	0.02	Non-IL-13
68	NFSQILPDPSKPSKR	0.01	Non-IL-13	0.01	Non-IL-13
	**AVERAGE**	**0.424**		**0.389**	

All the peptides having a score of > = 0.06 are considered to be IL-13 inducing. The higher values are highlighted in bold

*Indicates the same score for both tools

### Case study 4: re-construction of the improved IL-13 prediction classifier by enhanced training dataset

In order to have a more robust approach and accurate model, we increased positive training datasets by including additional experimentally validated human IL-13-inducing peptides obtained from the immune epitope database [20]. After removing the duplicates and retaining the peptides ranging from 8 to 35 amino acids, we were able to include 54 additional peptides in our positive dataset. The Pfeature algorithm was used to compute 9151 features of each peptide sequence. We then used the mRMR feature selection method to identify the most discriminatory features and performed experiments only with the top 10 and 95 features to build the machine learning models to be consistent with the earlier baseline experiments [19]. With this dataset, we report an increase in the performance as tabulated in Table 6.

**Table 6:** Comparison of the proposed model on the baseline dataset and the enhanced dataset on the basis of performance metrics

Classifier	Number of features selected	Sensitivity		Specificity		Accuracy		AUCROC		MCC	
		iIL13 Pred	iIL13Pred with enhanced positive dataset	iIL13 Pred	iIL13Pred with enhanced positive dataset	iIL13 Pred	iIL13Pred with enhanced positive dataset	iIL13 Pred	iIL13Pred with enhanced positive dataset	iIL13 Pred	iIL13Pred with enhanced positive dataset
RF	95	74.6	**76.19**	79.21	**79.89**	78.76	**79.53**	0.84	**0.86**	0.36	**0.38**
XGB	10	74.6	**76.19**	75.77	**77.66**	75.66	**77.51**	**0.83**	0.82	0.33	**0.35**

Random forest used 95 features and eXtreme gradient boosting used 10 features selected using mRMR. The higher values are highlighted in bold

## Discussion

IL-13 is shown to play a critical role in various biological processes. Several anti-IL-13 drugs for the cure of asthma and atopic dermatitis are in clinical trials [22–24]. Thus identification and characterization of novel drug molecules that can regulate IL-13 induction form an important area of research. Peptide-based drugs are rapidly becoming attractive due to their high specificity and low toxicity [25, 26]. Currently, there are more than a dozen peptide-based drug candidates that are in clinical trials [25]. Therefore extensive efforts are being put up into the prediction and validation of IL-13-inducing peptides. The prediction of IL-13-inducing peptides was taken up by a group recently and a tool named IL13Pred was published [19]. The positive and negative datasets included experimentally validated IL-13-inducing and non-inducing peptides respectively. The study used the Pfeature algorithm to compute the features. Feature selection was performed by the SVC-L1 algorithm and an appropriate library from the python script was used for feature ranking. Seven machine learning classifiers i.e. Decision Tree, Gaussian Naïve Bayes, *k*-Nearest Neighbour, Logistic Regression, Support Vector Machine, Random Forest, and eXtreme Gradient Boosting were then used to classify IL-13-inducing peptides. It was observed that among the seven machine learning classifiers, the best parameters were obtained using RF on the top 95 features whereas XGB performed best on the top 10 features. A user-friendly web server based on the XGB classifier was further developed. In an effort to improve the efficacy of the prediction tool, in this work, we have introduced an effective feature selection method that selects relevant and non-redundant features for building an improved decision model.

Selection of an appropriate feature selection is an important and critical pre-requisite step for model building, especially for biological data that is usually heterogeneous and of and high dimension [27]. The mRMR feature selection tool has been shown to select optimal and highly discriminatory features. It has been successfully used to select optimal features from the microarray datasets [28]. Radovic et al. incorporated mRMR to select more discriminative features in multivariate temporal gene expression datasets [29]. The potential of this tool was harnessed in building the prediction model of ovarian cancer survival [30]. Therefore, we sought to further improve the IL-13 prediction using a mRMR feature selection method.

In this study, extensive benchmarking was performed from the dataset obtained from the current study [19]. Following a similar procedure, features related to compositional descriptors were obtained from the Pfeature algorithm and seven common machine learning classifiers were used. The important contribution of our paper is the incorporation of the mRMR feature selection method and its effective performance on the experimentally validated dataset. In this study, we used the mRMR feature selection method, as compared to the current method (IL13Pred) that used SVC-L1 for the same. All the experiments were performed on top 10, 20, 30,…95 features, and the results obtained were compared with that of IL13Pred. Jain et al. [19] reported that with the top 95 features, the RF classifier outperformed the other classifiers with an AUCROC of 0.83 for the validation dataset. We show an improved AUCROC of 0.84 in the validation dataset. Additionally, our experiments resulted in an improved MCC of 0.36 on the validation dataset as compared to 0.34 from the existing method (IL13Pred). Jain et al. [19] further report that the XGB classifier with 10 features performed better with AUCROC of 0.83 and 0.80 on training and validation data respectively. We obtained an AUCROC of 0.84 and 0.83 on training and validation data with top 10 features thereby outperforming the existing method. We further report an improved MCC of 0.34 and 0.33 on training and validation datasets respectively as compared to 0.33 (training data) and 0.30 (validation data) reported by Jain et al. [19].

Although the existing tool was giving higher probability of IL-13-inducing peptides obtained from SARS-CoV-2 spike protein, however, the peptides used for the prediction were not experimentally validated (Case study 1 and 2). Therefore, to benchmark both tools, we obtained experimentally validated IL-13-inducing peptides from the immune epitope database. We show that our tool identified IL-13-inducing peptides with greater average prediction probability in comparison to the existing method (Case study 3). The XGB decision model with top 10 features performed better with the independent dataset. The strength of XGB decision model has also been demonstrated in the prediction of the bioactive molecules [31]. Jeon et al. illustrated the strengths of the AdaBoost baseline models in the final prediction of cellular localization of long non-coding RNAs (lncRNAs) [32].

To build a strong classifier we captured experimentally validated human IL-13-inducing peptides from the updated immune epitope database in a positive training dataset. Feature generation and feature selection were executed by the pfeature algorithm and mRMR selection tool. XGB model and RF classifiers were built on top 10 and 95 features. Performance metrics of the classifier were found to be better here as compared to our baseline approach (Case study 4).

We implemented our results in the form of a web server to enable researchers to predict potential IL-13-inducing peptides for experimental validation. User can predict whether a particular peptide can induce IL-13, further they can also generate different mutant versions of a particular peptide sequence and test their IL-13-inducing ability. In addition, the web server also allows the user to generate all the possible overlapping peptides of a particular protein and to predict their IL-13-inducing ability.

Several studies have utilized the properties of integrative machine learning frameworks in generating prediction models. Recent studies on the prediction of epigenetic modifications including DNA N6- methyladenine sites across several plant species indicate the potential of machine learning algorithms across plants and animal species [33, 34].

A limitation of this study is the availability of only a minuscule number of experimentally validated IL-13-inducing peptides. In the future, we shall work with diverse species for the discrimination between IL-13-inducing and non-inducing peptides. The availability of large datasets can also fuel the development of deep-learning models that identify patterns among IL-13-inducing peptides.

## Conclusions

IL-13 has been shown to be associated with the severity of several infectious diseases. Thus, the identification and characterization of novel IL-13-inducing molecules might lead to novel therapeutics. A recent study employed machine learning algorithms to classify IL-13-inducing peptides [19]. The present study was designed to further improve the prediction of IL-13 peptides by including an effective feature selection method that selects the most relevant and non-redundant feature set. We also obtained high performance in an additional experimentally validated IL-13-inducing peptide dataset. The most efficient model is integrated with a user-friendly web server to enable scientists to predict the IL-13-inducing potential of the peptides of interest which can then be tested experimentally.

## Supplementary Information

**Additional file 1**: **Table S1**. List of descriptors with a brief description and number of features computed using Pfeature algorithm.

**Additional file 2**: **Table S3**. Parameter range of seven machine learning models used in iIL13Pred for prediction of IL-13 inducing and non-inducing peptides.

**Additional file 3**: **Table S2**. Performance metrics of seven machine learning models for prediction of IL-13 inducing peptides on the top10 to top 95 features via mRMR (iIL13Pred) and its comparison with the state of the art (IL13Pred). The higher values are highlighted in bold.

**Additional file 4**: **Fig. S1**. Comparison of the averages of all the performance measures of the features (10–95) for various ML parameters on trainingand validation datasets for **A** Decision Tree training **B** Decision Tree validation **C** Gaussian Naive Bayes training **D** Gaussian Naive Bayes validation **E** *k*-Nearest Neighbour training **F** *k*-Nearest Neighbour validation **G** Logistic Regression training **H** Logistic Regression validation **I** Support Vector Machine training **J** Support Vector Machine validation

## Data Availability

The positive and negative datasets used in the training and testing of the models can be downloaded from https://webs.iiitd.edu.in/raghava/il13pred/dataset.php.

## Abbreviations

IL: Interleukin
IL13Pred: IL13 prediction
iIL13Pred: Improved IL13 prediction
mRMR: Minimum redundancy maximum relevance
DT: Decision tree
GNB: Gaussian NaïveBayes
KNN: k-Nearest Neighbour
LR: Logistic regression
SVM: Support vector machine
RF: Random forest
XGB: eXtreme gradient boosting
AUCROC: Area under the curve - receiver operating characteristics
MCC: Matthews correlation coefficient
COVID-19: Coronavirus disease 2019
Th-2: T-helper 2
ML: Machine learning
AWS: Amazon web service
HTML: Hyper text markup language
CSS: Cascading style sheets
TP: True positive
FP: False positive
TN: True negative
FN: False negative
SARS-CoV-2: Severe acute respiratory syndrome coronavirus -2
ROC: Receiver operating characteristics
IEDB: Immune epitope database
BTC_S: Composition of single bonds
CeTD_SS1: Composition of group 1 residues for secondary structure attribute
TPC_RQF: Composition of Arginine–Glutamine–PhenylAlanine
SER_I: Shannon entropy of isoleucine
SER_L: Shannon entropy of leucine
SER_T: Shannon entropy of threonine
BTC_T: Composition of total bonds
CeTD_75_p_HB1: Number of group 1 residues for hydrophobicity present in 75% quartile
AAC_H: Amino acid composition of histidine
SER_P: Shannon entropy of proline
SE: Shannon-entropy of protein
SOCN: Sequence order coupling number
ABC: Atomic and bond composition
AAC: Amino acid composition
DDOR: Distance distribution of residue
RRI: Residue repeat information
SER: Shannon entropy of all amino acids
PAAC: Pseudo amino acid composition
SEP: Shannon-entropy of physiochemical property
APAAC: Amphiphilic pseudo amino acid composition
QSO: Quasi-sequence order
CeTD: Composition-enhanced transition distribution
CTD: Conjoint triad calculation of the descriptor
DPC: Dipeptide composition
TPC: Tripeptide composition
