# Supplementary material for: iIL13Pred: improved prediction of IL-13 inducing peptides using popular machine learning classifiers
Source: BMC Bioinformatics. 2023 Apr 11;24:141. doi: 10.1186/s12859-023-05248-6 (PMC10088697; doi:10.1186/s12859-023-05248-6)
Supplement: Supplementary file 1 — Additional file 1: Table S1. List of descriptors with a brief description and number of features computed using Pfeature algorithm. [file 12859_2023_5248_MOESM1_ESM.docx]

Supplementary table 1: List of descriptors with a brief description and number of features computed using Pfeature algorithm.

| Name of descriptor | Description of descriptor | Number of features |
| --- | --- | --- |
| SE | Shannon-entropy of protein | 1 |
| SOCN | Sequence order coupling number | 6 |
| ABC | Atomic and bond composition | 9 |
| AAC | Amino acid composition | 20 |
| DDOR | Distance distribution of residue | 20 |
| RRI | Residue repeat Information | 20 |
| SER | Shannon entropy of all amino acids | 20 |
| PAAC | Pseudo amino acid composition | 23 |
| SEP | Shannon-entropy of physiochemical property | 25 |
| APAAC | Amphiphilic pseudo amino acid composition | 29 |
| QSO | Quasi-sequence order | 46 |
| CeTD | Composition-enhanced transition distribution | 189 |
| CTD | Conjoint triad calculation of the descriptor | 343 |
| DPC | Dipeptide Composition | 400 |
| TPC | Tripeptide Composition | 8000 |
|  | Total | 9151 |
