# Supplementary material for: iIL13Pred: improved prediction of IL-13 inducing peptides using popular machine learning classifiers
Source: BMC Bioinformatics. 2023 Apr 11;24:141. doi: 10.1186/s12859-023-05248-6 (PMC10088697; doi:10.1186/s12859-023-05248-6)
Supplement: Supplementary file 2 — Additional file 2: Table S3. Parameter range of seven machine learning models used in iIL13Pred for prediction of IL-13 inducing and non-inducing peptides. [file 12859_2023_5248_MOESM2_ESM.docx]

Supplementary Table 2: Parameter range of seven machine learning models used in iIL13Pred for prediction of IL-13 inducing and non-inducing peptides

| **Machine learning classifier** | **Parameter** |
| --- | --- |
| Decision Tree (DT) | criterion = gini or entropy; max_depth ranges from 10 to 40; max_features = log2 or sqrt; min_sample_split ranges 10 to 50 |
| Random Forest (RF) | criterion = entropy; max_features = auto; min_sample_split ranges from 5 to 40; max_depth ranges from 10 to 40; n_estimators ranges from 80 to 200 |
| Support Vector Machine (SVM) | kernel = rbf; gamma ranges from 0.01 to 0.1; C ranges from 10 to 100 |
| Gaussian Naïve Bayes (GNB) | prior ranges from 10^-4^ to 10^-64^ |
| *k* Nearest Neighbor (*K*NN) | weight = distance; p = 1 or 2; n_neighbors ranges from 4 to 8 |
| Logistic Regression (LR) | C ranges from 0.01 to 0.1 |
| eXtreme Gradient Boosting (XGB) | objective = binary:logistic; n_estimators = 80 to 200; max_depth ranges from 10 to 40; reg_lambda ranges from 1 to 20; eta ranges from 0.05 to 0.15 |
