# Supplementary material for: iIL13Pred: improved prediction of IL-13 inducing peptides using popular machine learning classifiers
Source: BMC Bioinformatics. 2023 Apr 11;24:141. doi: 10.1186/s12859-023-05248-6 (PMC10088697; doi:10.1186/s12859-023-05248-6)
Supplement: Supplementary file 3 — Additional file 3: Table S2. Performance metrics of seven machine learning models for prediction of IL-13 inducing peptides on the top10 to top 95 features via mRMR (iIL13Pred) and its comparison with the state of the art (IL13Pred). The higher values are highlighted in bold. [file 12859_2023_5248_MOESM3_ESM.docx]

Supplementary Table 3: Performance metrics of seven machine learning models for prediction of IL-13 inducing peptides on the top10 to top 95 features via mRMR (iIL13Pred) and its comparison with the state of the art (IL13Pred). The higher values are highlighted in bold.

| Top 10 Features | | | | | | | | | | | | |
| --- | --- | --- | --- | --- | --- | --- | --- | --- | --- | --- | --- | --- |
|  |  | **Threshold** | **Sensitivity** | | **Specificity** | | **Accuracy** | | **AUCROC** | | **MCC** | |
|  |  |  | **Present study** | **Jain et al.** | **Present study** | **Jain et al.** | **Present study** | **Jain et al.** | **Present study** | **Jain et al.** | **Present study** | **Jain et al.** |
| **DT** | **Training** | 0.05 | 64.8 | **69.36** | **72.18** | 69.46 | **71.47** | 69.45 | 0.72 | **0.74** | 0.24 | 0.24 |
|  | **Validation** | 0.05 | **65.08** | 60.26 | **76.29** | 71.43 | **75.19** | 70.08 | **0.75** | 0.72 | **0.27** | 0.22 |
| **GNB** | **Training** | 0.06 | **72** | 71.06 | **70.98** | 68.18 | **71.08** | 68.44 | **0.79** | 0.74 | **0.29** | 0.24 |
|  | **Validation** | 0.06 | **68.25** | 64.1 | **72.34** | 66.31 | **71.94** | 66.05 | **0.75** | 0.73 | **0.26** | 0.21 |
| ***k*NN** | **Training** | 0.07 | **68.4** | 65.11 | **66.17** | 56.47 | **66.38** | 57.26 | **0.72** | 0.64 | **0.21** | 0.13 |
|  | **Validation** | 0.07 | **65.08** | 60.26 | **69.93** | 55.73 | **69.46** | 56.28 | **0.7** | 0.61 | **0.22** | 0.11 |
| **LR** | **Training** | 0.09 | **66** | 64.26 | **62.59** | 61.85 | **62.92** | 62.07 | **0.67** | 0.67 | **0.17** | 0.15 |
|  | **Validation** | 0.09 | **60.32** | 56.41 | **62.03** | 59.61 | **61.86** | 59.23 | **0.63** | 0.63 | **0.14** | 0.11 |
| **RF** | **Training** | 0.09 | **76.4** | 74.47 | 75.02 | **76.34** | 75.16 | **76.17** | 0.84 | 0.83 | **0.34** | 0.33 |
|  | **Validation** | 0.09 | **68.25** | 64.1 | **78.18** | 75.13 | **77.21** | 73.8 | **0.8** | 0.77 | **0.31** | 0.28 |
| **XGB** | **Training** | 0.06 | **79.6** | 78.3 | 73.99 | **74.84** | 74.53 | **75.16** | **0.84** | 0.83 | **0.34** | 0.33 |
|  | **Validation** | 0.06 | **74.6** | 71.8 | **75.77** | 73.02 | **75.66** | 72.87 | **0.83** | 0.8 | **0.33** | 0.3 |
| **SVC** | **Training** | 0.09 | **60.4** | 54.47 | **74.46** | 70.7 | **73.1** | 69.22 | **0.74** | 0.67 | **0.23** | 0.16 |
|  | **Validation** | 0.09 | **49.21** | 46.15 | **79.04** | 74.25 | **76.12** | 70.85 | **0.71** | 0.64 | **0.2** | 0.15 |

| Top 20 Features | | | | | | | | | | | | |
| --- | --- | --- | --- | --- | --- | --- | --- | --- | --- | --- | --- | --- |
|  |  | **Threshold** | **Sensitivity** | | **Specificity** | | **Accuracy** | | **AUCROC** | | **MCC** | |
|  |  |  | **Present study** | **Jain et al.** | **Present study** | **Jain et al.** | **Present study** | **Jain et al.** | **Present study** | **Jain et al.** | **Present study** | **Jain et al.** |
| **DT** | **Training** | 0.07 | **68.4** | 67.66 | **70.29** | 68.005 | **70.11** | 67.974 | **0.742** | 0.716 | **0.245** | 0.215 |
|  | **Validation** | 0.07 | **71.43** | 70.513 | **69.59** | 64.021 | **69.77** | 64.806 | **0.724** | 0.739 | **0.256** | 0.23 |
| **GNB** | **Training** | 0.31 | **67.2** | 66.383 | **75.19** | 66.425 | **74.42** | 66.421 | **0.79** | 0.724 | **0.298** | 0.196 |
|  | **Validation** | 0.31 | **58.73** | 50 | **75.26** | 73.369 | **73.64** | 70.543 | **0.758** | 0.682 | **0.224** | 0.167 |
| ***k*NN** | **Training** | 0.08 | **68.4** | 57.447 | **67.03** | 63.691 | **67.16** | 63.121 | **0.738** | 0.634 | **0.218** | 0.125 |
|  | **Validation** | 0.08 | **68.25** | 58.974 | **68.9** | 68.078 | **68.84** | 66.977 | **0.745** | 0.637 | **0.232** | 0.185 |
| **LR** | **Training** | 0.093 | **64.4** | 63.83 | **66.294** | 64.63 | **66.11** | 64.557 | **0.693** | 0.698 | **0.189** | 0.169 |
|  | **Validation** | 0.093 | 63.49 | 64.103 | **65.29** | 60.494 | **65.12** | 60.93 | 0.655 | **0.677** | **0.176** | 0.162 |
| **RF** | **Training** | 0.12 | **76** | 75.745 | **76.52** | 76.42 | **76.47** | 76.359 | 0.823 | **0.842** | **0.346** | 0.333 |
|  | **Validation** | 0.12 | **76.19** | 67.949 | **75.95** | 75.132 | **76.9** | 74.264 | **0.825** | 0.82 | **0.341** | 0.306 |
| **XGB** | **Training** | 0.05 | 71.2 | 76.17 | **76.57** | 73.857 | **76.05** | 74.068 | 0.806 | **0.83** | **0.32** | 0.312 |
|  | **Validation** | 0.05 | **74.6** | 67.949 | **78.01** | 71.429 | **77.67** | 71.008 | **0.845** | 0.793 | **0.351** | 0.272 |
| **SVC** | **Training** | 0.09 | **64.4** | 56.596 | **74.68** | 69.628 | **73.68** | 68.439 | **0.75** | 0.678 | **0.255** | 0.161 |
|  | **Validation** | 0.09 | **61.9** | 47.436 | **77.66** | 73.192 | **76.12** | 70.078 | **0.769** | 0.649 | **0.267** | 0.148 |

| Top 30 features | | | | | | | | | | | | |
| --- | --- | --- | --- | --- | --- | --- | --- | --- | --- | --- | --- | --- |
|  |  | **Threshold** | **Sensitivity** | | **Specificity** | | **Accuracy** | | **AUCROC** | | **MCC** | |
|  |  |  | **Present study** | **Jain et al.** | **Present study** | **Jain et al.** | **Present study** | **Jain et al.** | **Present study** | **Jain et al.** | **Present study** | **Jain et al.** |
| **DT** | **Training** | 0.07 | **66.8** | 64.255 | **72.14** | 64.63 | **71.62** | 64.596 | **0.721** | 0.683 | **0.254** | 0.171 |
|  | **Validation** | 0.07 | **68.25** | 61.538 | **73.88** | 63.668 | **73.33** | 63.411 | **0.738** | 0.673 | **0.272** | 0.168 |
| **GNB** | **Training** | 0.43 | **70.4** | 69.362 | **70.04** | 69.201 | **70.07** | 69.216 | **0.802** | 0.74 | **0.269** | 0.234 |
|  | **Validation** | 0.43 | **66.67** | 55.128 | **74.05** | 72.663 | **73.33** | 70.543 | **0.76** | 0.68 | **0.264** | 0.196 |
| ***k*NN** | **Training** | 0.07 | **69.2** | 62.979 | **69.18** | 55.959 | **69.18** | 56.599 | **0.747** | 0.628 | **0.239** | 0.109 |
|  | **Validation** | 0.07 | **68.25** | 62.821 | **72.68** | 55.203 | **72.25** | 56.124 | **0.753** | 0.619 | **0.262** | 0.118 |
| **LR** | **Training** | 0.09 | **67.2** | 65.532 | 65 | 67.151 | 65.21 | 67.003 | 0.706 | **0.727** | 0.196 | 0.196 |
|  | **Validation** | 0.09 | **63.49** | 62.821 | **65.12** | 62.61 | **64.96** | 62.636 | **0.681** | 0.68 | **0.175** | 0.169 |
| **RF** | **Training** | 0.12 | 76.4 | 77.447 | 76.31 | 79.197 | 76.32 | 79.037 | 0.826 | **0.851** | 0.345 | **0.372** |
|  | **Validation** | 0.12 | **73.02** | 70.513 | **80.58** | 77.425 | **79.84** | 76.589 | **0.834** | 0.814 | **0.369** | 0.347 |
| **XGB** | **Training** | 0.06 | 73.2 | 77.447 | **75.41** | 75.139 | 75.19 | 75.349 | 0.827 | **0.843** | 0.317 | **0.331** |
|  | **Validation** | 0.06 | **71.43** | 67.949 | **78.69** | 71.605 | **77.98** | 71.163 | **0.83** | 0.803 | **0.33** | 0.274 |
| **SVC** | **Training** | 0.09 | **64** | 55.745 | **73.26** | 69.885 | **72.36** | 68.595 | **0.764** | 0.671 | **0.24** | 0.158 |
|  | **Validation** | 0.09 | **60.32** | 50 | **75.6** | 72.84 | **74.11** | 70.078 | **0.749** | 0.65 | **0.238** | 0.163 |

| Top 40 features | | | | | | | | | | | | |
| --- | --- | --- | --- | --- | --- | --- | --- | --- | --- | --- | --- | --- |
|  |  | **Threshold** | **Sensitivity** | | **Specificity** | | **Accuracy** | | **AUCROC** | | **MCC** | |
|  |  |  | **Present study** | **Jain et al.** | **Present study** | **Jain et al.** | **Present study** | **Jain et al.** | **Present study** | **Jain et al.** | **Present study** | **Jain et al.** |
| **DT** | **Training** | 0.07 | **65.6** | 64.255 | **71.02** | 63.99 | **70.5** | 64.014 | **0.701** | 0.67 | **0.234** | 0.167 |
|  | **Validation** | 0.07 | **73.02** | 71.795 | **68.56** | 55.026 | **68.99** | 57.054 | **0.73** | 0.68 | **0.258** | 0.175 |
| **GNB** | **Training** | 0.2 | **73.2** | 71.489 | **69.77** | 68.091 | **70.11** | 68.401 | **0.8** | 0.755 | **0.282** | 0.238 |
|  | **Validation** | 0.2 | **63.49** | 51.282 | **73.19** | 73.898 | **72.25** | 71.163 | **0.76** | 0.68 | **0.24** | 0.181 |
| ***k*NN** | **Training** | 0.08 | **71.6** | 57.447 | **66.98** | 64.716 | **67.43** | 64.053 | **0.759** | 0.631 | **0.237** | 0.132 |
|  | **Validation** | 0.08 | **73.02** | 52.564 | **73.37** | 67.019 | **73.33** | 65.271 | **0.783** | 0.624 | **0.297** | 0.134 |
| **LR** | **Training** | 0.085 | 68.8 | 68.936 | 67.32 | 67.663 | 67.46 | 67.78 | 0.724 | **0.753** | 0.222 | 0.22 |
|  | **Validation** | 0.085 | 65.08 | 66.667 | 62.89 | 61.023 | 63,10 | 61.705 | 0.674 | **0.694** | 0.17 | **0.183** |
| **RF** | **Training** | 0.12 | 74.4 | 77.872 | 76.31 | 76.335 | 76.12 | 76.475 | 0.822 | **0.856** | 0.333 | **0.345** |
|  | **Validation** | 0.12 | **74.6** | 71.795 | **81.44** | 75.838 | **80.77** | 75.349 | **0.835** | 0.828 | **0.389** | 0.339 |
| **XGB** | **Training** | 0.06 | **76** | 75.745 | 72.14 | 77.531 | 72.51 | 77.368 | 0.815 | **0.85** | 0.307 | **0.344** |
|  | **Validation** | 0.06 | **71.43** | 61.538 | **81.79** | 77.249 | **80.78** | 75.349 | **0.837** | 0.794 | **0.373** | 0.283 |
| **SVC** | **Training** | 0.07 | **72.8** | 66.809 | **67.28** | 64.502 | **67.82** | 64.713 | **0.789** | 0.706 | **0.247** | 0.185 |
|  | **Validation** | 0.07 | **71.43** | 53.846 | **69.59** | 62.081 | **69.77** | 61.085 | **0.784** | 0.636 | **0.256** | 0.106 |

| Top 50 features | | | | | | | | | | | | |
| --- | --- | --- | --- | --- | --- | --- | --- | --- | --- | --- | --- | --- |
|  |  | **Threshold** | **Sensitivity** | | **Specificity** | | **Accuracy** | | **AUCROC** | | **MCC** | |
|  |  |  | **Present study** | **Jain et al.** | **Present study** | **Jain et al.** | **Present study** | **Jain et al.** | **Present study** | **Jain et al.** | **Present study** | **Jain et al.** |
| **DT** | **Training** | 0.07 | **69.2** | 55.745 | **70.08** | 70.226 | **69.99** | 68.905 | **0.744** | 0.66 | **0.247** | 0.16 |
|  | **Validation** | 0.07 | **69.84** | 56.41 | **74.74** | 69.489 | **74.26** | 67.907 | **0.749** | 0.66 | **0.29** | 0.179 |
| **GNB** | **Training** | 0.95 | **71.2** | 70.638 | **74.33** | 68.261 | **74.03** | 68.478 | **0.804** | 0.752 | **0.308** | 0.234 |
|  | **Validation** | 0.95 | **63.49** | 51.282 | **73.2** | 71.076 | **72.25** | 68.682 | **0.755** | 0.663 | **0.237** | 0.157 |
| ***k*NN** | **Training** | 0.08 | **61.2** | 59.149 | **78.2** | 64.417 | **76.55** | 63.936 | **0.739** | 0.641 | **0.267** | 0.14 |
|  | **Validation** | 0.08 | **73.02** | 56.41 | **81.79** | 68.078 | **80.93** | 66.667 | **0.809** | 0.621 | **0.383** | 0.168 |
| **LR** | **Training** | 0.086 | 69.2 | 69.787 | 68.01 | 71.081 | 68.13 | 70.963 | 0.735 | **0.764** | 0.23 | **0.251** |
|  | **Validation** | 0.086 | **60.32** | 58.974 | **64.78** | 64.55 | **64.34** | 63.876 | **0.681** | 0.678 | **0.159** | 0.158 |
| **RF** | **Training** | 0.12 | 76.8 | 77.872 | 75.97 | 78.983 | 76.05 | 78.882 | 0.828 | **0.862** | 0.343 | **0.372** |
|  | **Validation** | 0.12 | **74.6** | 69.231 | **81.1** | 79.365 | **80.46** | 78.14 | **0.839** | 0.831 | **0.385** | 0.359 |
| **XGB** | **Training** | 0.06 | **75.2** | 74.468 | 71.88 | 75.95 | 72.2 | 75.815 | 0.798 | **0.844** | 0.301 | **0.321** |
|  | **Validation** | 0.06 | **68.25** | 62.821 | **78.52** | 73.192 | **77.52** | 71.938 | **0.811** | 0.787 | **0.316** | 0.254 |
| **SVC** | **Training** | 0.07 | **70.4** | 69.787 | **70.34** | 67.493 | **70.38** | 67.702 | **0.794** | 0.736 | **0.255** | 0.224 |
|  | **Validation** | 0.07 | **71.43** | 58.974 | **71.82** | 66.667 | **71.78** | 65.736 | **0.773** | 0.642 | **0.274** | 0.174 |

| Top 60 Features | | | | | | | | | | | | |
| --- | --- | --- | --- | --- | --- | --- | --- | --- | --- | --- | --- | --- |
|  |  | **Threshold** | **Sensitivity** | | **Specificity** | | **Accuracy** | | **AUCROC** | | **MCC** | |
|  |  |  | **Present study** | **Jain et al.** | **Present study** | **Jain et al.** | **Present study** | **Jain et al.** | **Present study** | **Jain et al.** | **Present study** | **Jain et al.** |
| **DT** | **Training** | 0.08 | **62.4** | 57.447 | **75.44** | 72.832 | **74.17** | 71.429 | **0.705** | 0.687 | **0.258** | 0.19 |
|  | **Validation** | 0.08 | **61.9** | 43.59 | **78.87** | 73.721 | **77.21** | 70.078 | **0.73** | 0.62 | **0.279** | 0.125 |
| **GNB** | **Training** | 0.99 | 72 | **74.468** | **68.48** | 64.972 | **68.82** | 65.839 | **0.8** | 0.759 | **0.283** | 0.233 |
|  | **Validation** | 0.99 | **﻿65.08** | 56.41 | **69.24** | 64.021 | **68.84** | 63.101 | **0.758** | 0.63 | **0.215** | 0.137 |
| ***k*NN** | **Training** | 0.09 | **70** | 57.021 | **74.98** | 64.203 | **74.5** | 63.548 | **0.771** | 0.641 | **0.292** | 0.126 |
|  | **Validation** | 0.09 | **71.43** | 57.692 | **76.98** | 67.372 | **76.43** | 66.202 | **0.789** | 0.623 | **0.321** | 0.171 |
| **LR** | **Training** | 0.085 | 67.6 | 71.064 | 69.17 | 70.867 | 69.02 | 70.885 | 0.757 | **0.777** | 0.229 | **0.257** |
|  | **Validation** | 0.085 | **61.9** | 60.256 | **67.01** | 62.963 | **66.51** | 62.636 | **0.7** | 0.654 | **0.179** | 0.155 |
| **RF** | **Training** | 0.12 | 75.2 | 77.872 | 77.04 | 78.855 | 76.86 | 78.766 | 0.84 | **0.864** | 0.348 | **0.371** |
|  | **Validation** | 0.12 | **73.02** | 69.231 | **81.1** | 77.954 | **80.31** | 76.899 | **0.842** | 0.818 | **0.375** | 0.344 |
| **XGB** | **Training** | 0.06 | 73.2 | 77.872 | 75.5 | 76.677 | 75.27 | 76.786 | 0.813 | **0.845** | 0.319 | **0.349** |
|  | **Validation** | 0.06 | **69.84** | 66.667 | **80.58** | 72.663 | **79.53** | 71.938 | **0.817** | 0.796 | **0.349** | 0.275 |
| **SVC** | **Training** | 0.08 | **70.4** | 67.234 | **77.51** | 68.048 | **76.82** | 67.974 | **0.823** | 0.745 | **0.319** | 0.213 |
|  | **Validation** | 0.08 | **65.08** | 46.154 | **78.35** | 64.198 | **77.05** | 62.016 | **0.787** | 0.589 | **0.294** | 0.07 |

| Top 70 Features | | | | | | | | | | | | |
| --- | --- | --- | --- | --- | --- | --- | --- | --- | --- | --- | --- | --- |
|  |  | **Threshold** | **Sensitivity** | | **Specificity** | | **Accuracy** | | **AUCROC** | | **MCC** | |
|  |  |  | **Present study** | **Jain et al.** | **Present study** | **Jain et al.** | **Present study** | **Jain et al.** | **Present study** | **Jain et al.** | **Present study** | **Jain et al.** |
| **DT** | **Training** | 0.08 | **69.6** | 65.957 | **70.02** | 65.955 | **69.97** | 65.955 | **0.729** | 0.71 | **0.256** | 0.19 |
|  | **Validation** | 0.08 | 66.67 | 70.513 | **78.69** | 62.257 | **77.52** | 63.256 | **0.757** | 0.726 | **0.308** | 0.217 |
| **GNB** | **Training** | 0.9 | 73.2 | 74.043 | **66.6** | 64.759 | **67.24** | 65.606 | **0.8** | 0.76 | **0.279** | 0.229 |
|  | **Validation** | 0.9 | **68.25** | 56.41 | **62.54** | 61.905 | **63.1** | 61.24 | **0.735** | 0.613 | **0.186** | 0.122 |
| ***k*NN** | **Training** | **0.08** | **64.4** | 59.149 | **77.56** | 64.032 | **76.24** | 63.587 | **0.742** | 0.639 | **0.279** | 0.137 |
|  | **Validation** | **0.08** | **63.49** | 57.692 | **79.55** | 67.372 | **77.98** | 66.202 | **0.745** | 0.628 | **0.297** | 0.171 |
| **LR** | **Training** | 0.085 | 70 | 73.617 | 66.51 | 73.217 | 66.85 | 73.253 | 0.76 | **0.792** | 0.22 | 0.291 |
|  | **Validation** | 0.085 | **65.07** | 56.41 | **67.35** | 65.785 | **67.13** | 64.651 | **0.717** | 0.657 | **0.201** | 0.15 |
| **RF** | **Training** | 0.12 | 78.8 | 78.723 | 74.07 | 78.172 | 74.53 | 78.222 | 0.827 | **0.862** | 0.341 | **0.369** |
|  | **Validation** | 0.12 | **73.02** | 69.231 | **80.93** | 76.72 | **80.16** | 75.814 | **0.833** | 0.819 | **0.373** | 0.331 |
| **XGB** | **Training** | 0.05 | 72.8 | 77.447 | 75.71 | 76.89 | 75.43 | 76.941 | 0.811 | **0.848** | 0.319 | **0.348** |
|  | **Validation** | 0.05 | **74.6** | 71.795 | **77.32** | 70.899 | **77.05** | 71.008 | **0.821** | 0.785 | **0.344** | 0.293 |
| **SVC** | **Training** | 0.09 | 68.4 | 69.787 | **81.51** | 70.611 | **80.24** | 70.536 | **0.813** | 0.759 | **0.349** | 0.247 |
|  | **Validation** | 0.09 | **61.9** | 46.154 | **80.76** | 69.312 | **78.91** | 66.512 | **0.783** | 0.596 | **0.299** | 0.108 |

| Top 80 Features | | | | | | | | | | | | |
| --- | --- | --- | --- | --- | --- | --- | --- | --- | --- | --- | --- | --- |
|  |  | **Threshold** | **Sensitivity** | | **Specificity** | | **Accuracy** | | **AUCROC** | | **MCC** | |
|  |  |  | **Present study** | **Jain et al.** | **Present study** | **Jain et al.** | **Present study** | **Jain et al.** | **Present study** | **Jain et al.** | **Present study** | **Jain et al.** |
| **DT** | **Training** | 0.08 | **66.8** | 65.957 | **72.35** | 66.425 | **71.81** | 66.382 | **0.726** | 0.689 | **0.258** | 0.194 |
|  | **Validation** | 0.08 | **68.25** | 62.821 | **71.13** | 61.552 | **70.85** | 61.705 | **0.765** | 0.618 | **0.249** | 0.161 |
| **GNB** | **Training** | 0.85 | **76** | 71.489 | **67.8** | 64.545 | **68.59** | 65.179 | **0.805** | 0.758 | **0.277** | 0.213 |
|  | **Validation** | 0.85 | **68.25** | 51.282 | **63.92** | 61.905 | **64.34** | 60.62 | **0.74** | 0.612 | **0.196** | 0.088 |
| ***k*NN** | **Training** | 0.08 | **66.4** | 59.149 | **76.18** | 63.392 | **75.23** | 63.005 | **0.756** | 0.638 | **0.281** | 0.133 |
|  | **Validation** | 0.08 | **68.25** | 57.692 | **78.52** | 67.196 | **77.52** | 66.047 | **0.769** | 0.624 | **0.316** | 0.169 |
| **LR** | **Training** | 0.085 | 70 | 73.617 | 69 | 74.284 | 69.1 | 74.224 | 0.764 | **0.801** | 0.243 | 0.301 |
|  | **Validation** | 0.085 | **65.08** | 53.846 | **71.82** | 68.783 | **71.16** | 66.977 | **0.73** | 0.659 | **0.235** | 0.156 |
| **RF** | **Training** | 0.11 | 78 | 79.149 | 74.42 | 79.667 | 74.77 | 79.62 | 0.836 | **0.871** | 0.339 | **0.388** |
|  | **Validation** | 0.11 | **77.78** | 70.513 | **78** | 77.954 | **77.98** | 77.054 | **0.841** | 0.819 | **0.371** | 0.352 |
| **XGB** | **Training** | 0.05 | 71.6 | 76.17 | 74.85 | 76.207 | 74.53 | 76.203 | 0.806 | **0.836** | 0.304 | **0.334** |
|  | **Validation** | 0.05 | **69.84** | 67.949 | **75.77** | 71.429 | **75.19** | 71.008 | **0.815** | 0.798 | **0.299** | 0.272 |
| **SVC** | **Training** | 0.08 | **72.4** | 68.936 | **77.34** | 69.97 | **76.86** | 69.876 | **0.811** | 0.764 | **0.33** | 0.237 |
|  | **Validation** | 0.08 | **69.84** | 44.872 | **76.29** | 68.607 | **75.66** | 65.736 | **0.796** | 0.594 | **0.304** | 0.093 |

| Top 90 Features | | | | | | | | | | | | |
| --- | --- | --- | --- | --- | --- | --- | --- | --- | --- | --- | --- | --- |
|  |  | **Threshold** | **Sensitivity** | | **Specificity** | | **Accuracy** | | **AUCROC** | | **MCC** | |
|  |  |  | **Present study** | **Jain et al.** | **Present study** | **Jain et al.** | **Present study** | **Jain et al.** | **Present study** | **Jain et al.** | **Present study** | **Jain et al.** |
| **DT** | **Training** | 0.09 | 62.8 | 64.681 | **78.68** | 64.502 | **77.14** | 64.519 | **0.733** | 0.692 | **0.284** | 0.173 |
|  | **Validation** | 0.09 | **61.9** | 53.846 | **76.12** | 65.079 | **74.73** | 63.721 | **0.701** | 0.639 | **0.253** | 0.128 |
| **GNB** | **Training** | 0.1 | 66.8 | 72.34 | **78.89** | 72.533 | **77.72** | 72.516 | **0.801** | 0.782 | **0.32** | 0.278 |
|  | **Validation** | 0.1 | **55.56** | 48.718 | **72.34** | 70.37 | **70.7** | 67.752 | **0.72** | 0.622 | **0.18** | 0.133 |
| ***k*NN** | **Training** | 0.08 | **65.2** | 56.17 | **78.93** | 65.271 | **77.6** | 64.441 | **0.756** | 0.633 | **0.3** | 0.128 |
|  | **Validation** | 0.08 | **63.49** | 50 | **80.93** | 68.783 | **79.22** | 66.512 | **0.749** | 0.621 | **0.311** | 0.13 |
| **LR** | **Training** | 0.085 | 70 | 74.468 | 70.64 | 74.754 | 70.57 | 74.728 | 0.769 | **0.822** | 0.256 | **0.31** |
|  | **Validation** | 0.085 | **63.49** | 56.41 | **71.48** | 68.783 | **70.7** | 67.287 | **0.726** | 0.673 | **0.223** | 0.173 |
| **RF** | **Training** | 0.1 | 78.4 | 80.426 | 75.84 | 80.222 | 76.09 | 80.241 | 0.847 | **0.878** | 0.354 | **0.402** |
|  | **Validation** | 0.1 | **74.6** | 71.795 | **79.55** | 78.131 | **79.06** | 77.364 | **0.821** | 0.827 | **0.368** | 0.363 |
| **XGB** | **Training** | 0.05 | 72.4 | 78.298 | 76.74 | 77.83 | 76.32 | 77.873 | 0.821 | **0.852** | 0.327 | **0.363** |
|  | **Validation** | 0.05 | **68.25** | 66.667 | **82.99** | 78.483 | **81.55** | 77.054 | **0.829** | 0.811 | **0.367** | 0.332 |
| **SVC** | **Training** | 0.08 | **72** | 71.915 | **77.21** | 72.619 | **76.71** | 72.554 | **0.822** | 0.791 | **0.327** | 0.276 |
|  | **Validation** | 0.08 | **61.9** | 51.282 | **77.32** | 70.723 | **75.81** | 68.372 | **0.782** | 0.63 | **0.264** | 0.154 |

| Top 95 Features | | | | | | | | | | | | |
| --- | --- | --- | --- | --- | --- | --- | --- | --- | --- | --- | --- | --- |
|  |  | **Threshold** | **Sensitivity** | | **Specificity** | | **Accuracy** | | **AUCROC** | | **MCC** | |
|  |  |  | **Present study** | **Jain et al.** | **Present study** | **Jain et al.** | **Present study** | **Jain et al.** | **Present study** | **Jain et al.** | **Present study** | **Jain et al.** |
| **DT** | **Training** | 0.08 | **70.8** | 65.96 | **76.09** | 66.38 | **75.57** | 66.34 | **0.76** | 0.7 | **0.31** | 0.19 |
|  | **Validation** | 0.08 | **63.49** | 51.28 | **74.4** | 66.31 | **73.33** | 64.5 | **0.71** | 0.6 | **0.25** | 0.12 |
| **GNB** | **Training** | 0.15 | **67.12** | 63.83 | **80.13** | 79.58 | **78.88** | 78.14 | **0.81** | 0.78 | **0.33** | 0.29 |
|  | **Validation** | 0.15 | **50.79** | 38.46 | **78.69** | 77.43 | **75.97** | 72.71 | **0.71** | 0.61 | **0.2** | 0.12 |
| ***k*NN** | **Training** | 0.08 | **66** | 57.02 | **69.91** | 65.61 | **69.53** | 64.83 | **0.73** | 0.64 | **0.23** | 0.14 |
|  | **Validation** | 0.08 | **61.9** | 50 | **81.79** | 69.31 | **79.84** | 66.98 | **0.74** | 0.62 | **0.31** | 0.13 |
| **LR** | **Training** | 0.08 | 70 | **73.62** | 70 | **73.99** | 70 | **73.95** | 0.77 | **0.83** | 0.25 | 0.3 |
|  | **Validation** | 0.08 | **61.9** | 58.97 | **73.37** | 68.25 | **72.25** | 67.13 | **0.71** | 0.68 | **0.23** | 0.19 |
| **RF** | **Training** | 0.11 | 77.6 | **82.13** | 76.78 | **79.88** | 76.86 | **80.09** | 0.85 | **0.88** | 0.36 | **0.41** |
|  | **Validation** | 0.11 | **74.6** | 70.51 | **79.21** | 77.07 | **78.76** | 76.28 | **0.84** | 0.83 | **0.36** | 0.34 |
| **XGB** | **Training** | 0.04 | **77.27** | 73.62 | 72.27 | **76.59** | 72.76 | **76.32** | 0.83 | **0.84** | 0.31 | **0.32** |
|  | **Validation** | 0.04 | **73.02** | 69.23 | **79.73** | 73.19 | **79.07** | 72.71 | **0.81** | 0.8 | **0.36** | 0.3 |
| **SVC** | **Training** | 0.07 | **74** | 72.34 | **72.14** | 71.25 | **72.32** | 71.35 | **0.82** | 0.79 | **0.29** | 0.27 |
|  | **Validation** | 0.07 | **68.25** | 51.28 | **71.31** | 68.08 | **71** | 66.05 | **0.8** | 0.62 | **0.25** | 0.13 |
