# Supplementary figures and images for: iIL13Pred: improved prediction of IL-13 inducing peptides using popular machine learning classifiers
Source: BMC Bioinformatics. 2023 Apr 11;24:141. doi: 10.1186/s12859-023-05248-6 (PMC10088697; doi:10.1186/s12859-023-05248-6)

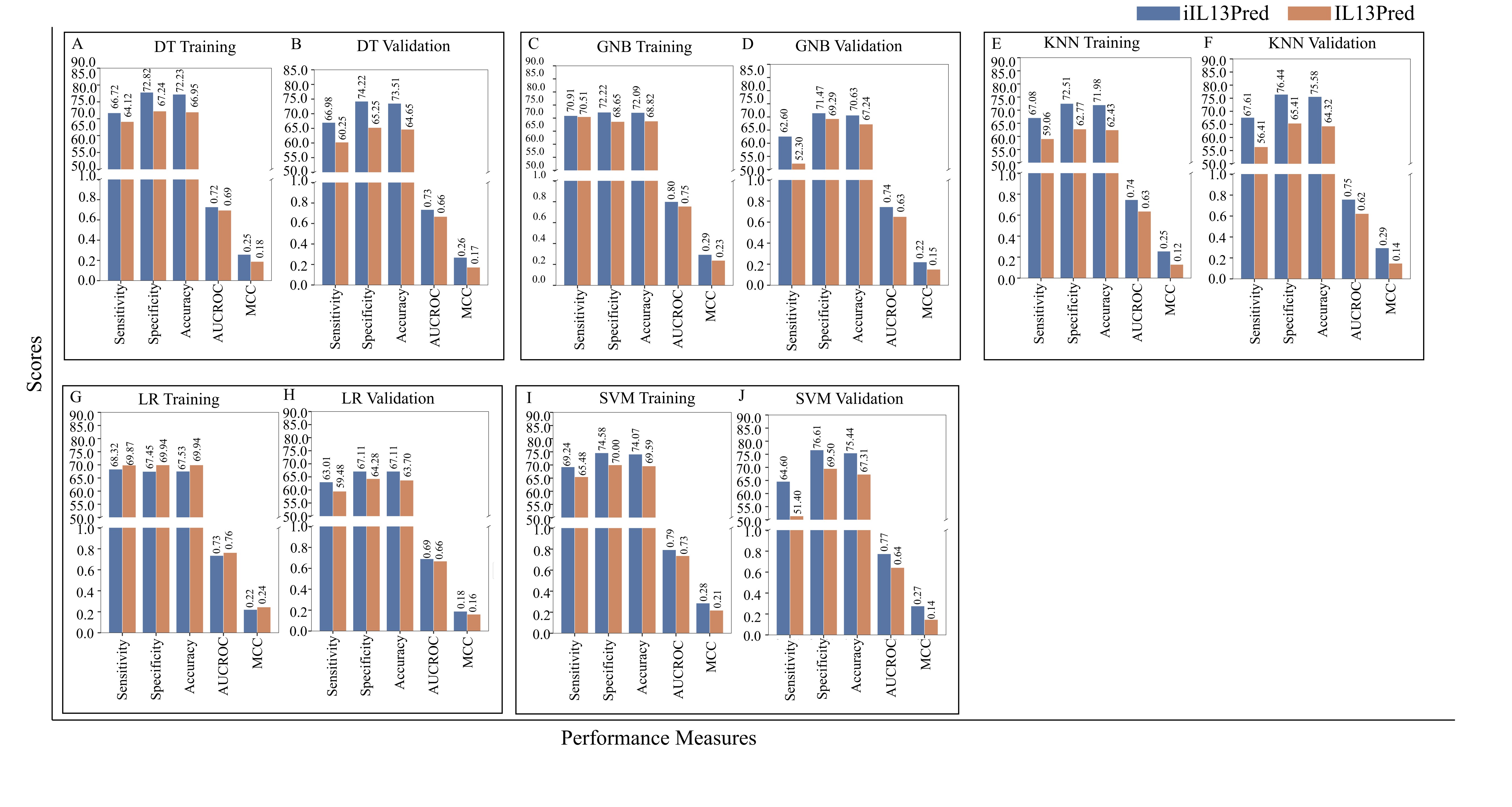

Supplement: Supplementary file 4 — Additional file 4: Fig. S1. Comparison of the averages of all the performance measures of the features (10–95) for various ML parameters on trainingand validation datasets for A Decision Tree training B Decision Tree validation C Gaussian Naive Bayes training D Gaussian Naive Bayes validation E k-Nearest Neighbour training F k-Nearest Neighbour validation G Logistic Regression training H Logistic Regression validation I Support Vector Machine training J Support Vector Machine validation [file 12859_2023_5248_MOESM4_ESM.jpg]
